# Risk factors for human papillomavirus infection, cervical intraepithelial neoplasia and cervical cancer: an umbrella review and follow-up Mendelian randomisation studies

**DOI:** 10.1186/s12916-023-02965-w

**Published:** 2023-07-27

**Authors:** Sarah J. Bowden, Triada Doulgeraki, Emmanouil Bouras, Georgios Markozannes, Antonios Athanasiou, Harriet Grout-Smith, Konstantinos S. Kechagias, Laura Burney Ellis, Verena Zuber, Marc Chadeau-Hyam, James M. Flanagan, Konstantinos K. Tsilidis, Ilkka Kalliala, Maria Kyrgiou

**Affiliations:** 1 Department of Metabolism, Digestion and Reproduction and Department of Surgery and Cancer, Institute of Reproductive and Developmental Biology, Faculty of Medicine, Imperial College London, Hammersmith Hospital campus, London, W12 0HS UK; 2 Queen Charlotte’s and Chelsea – Hammersmith Hospital, Imperial College Healthcare NHS Trust, London, UK; 3 Department of Hygiene and Epidemiology, University of Ioannina School of Medicine, Ioannina, Greece; 4 Department of Epidemiology and Biostatistics, School of Public Health, Imperial College London, London, UK; 5 Department of Surgery and Cancer, Institute of Reproductive and Developmental Biology, Faculty of Medicine, Imperial College London, London, UK; 6 Department of Obstetrics and Gynaecology, University of Helsinki and Helsinki University Hospital, Helsinki, Finland

**Keywords:** HPV, Cervical cancer, Cervical intraepithelial neoplasia, CIN, Umbrella, Mendelian randomisation, Microbiome

## Abstract

**Background:**

Persistent infection by oncogenic human papillomavirus (HPV) is necessary although not sufficient for development of cervical cancer. Behavioural, environmental, or comorbid exposures may promote or protect against malignant transformation. Randomised evidence is limited and the validity of observational studies describing these associations remains unclear.

**Methods:**

In this umbrella review, we searched electronic databases to identify meta-analyses of observational studies that evaluated risk or protective factors and the incidence of HPV infection, cervical intra-epithelial neoplasia (CIN), cervical cancer incidence and mortality. Following re-analysis, evidence was classified and graded based on a pre-defined set of statistical criteria. Quality was assessed with AMSTAR-2. For all associations graded as weak evidence or above, with available genetic instruments, we also performed Mendelian randomisation to examine the potential causal effect of modifiable exposures with risk of cervical cancer. The protocol for this study was registered on PROSPERO (CRD42020189995).

**Results:**

We included 171 meta-analyses of different exposure contrasts from 50 studies. Systemic immunosuppression including HIV infection (RR = 2.20 (95% CI = 1.89–2.54)) and immunosuppressive medications for inflammatory bowel disease (RR = 1.33 (95% CI = 1.27–1.39)), as well as an altered vaginal microbiome (RR = 1.59 (95% CI = 1.40–1.81)), were supported by strong and highly suggestive evidence for an association with HPV persistence, CIN or cervical cancer. Smoking, number of sexual partners and young age at first pregnancy were supported by highly suggestive evidence and confirmed by Mendelian randomisation.

**Conclusions:**

Our main analysis supported the association of systemic (HIV infection, immunosuppressive medications) and local immunosuppression (altered vaginal microbiota) with increased risk for worse HPV and cervical disease outcomes. Mendelian randomisation confirmed the link for genetically predicted lifetime smoking index, and young age at first pregnancy with cervical cancer, highlighting also that observational evidence can hide different inherent biases. This evidence strengthens the need for more frequent HPV screening in people with immunosuppression, further investigation of the vaginal microbiome and access to sexual health services.

**Supplementary Information:**

The online version contains supplementary material available at 10.1186/s12916-023-02965-w.

## Background

Although persistent infection with high-risk human papillomavirus (hrHPV) is causally associated with cervical cancer, only a fraction of women that get infected with HPV develop persistence, high-grade CIN and if not detected and treated cervical cancer. Cancer promotion in some individuals is likely to be explained by a complex interplay between the host system, the HPV virus and behavioural, environmental, or comorbid factors. Genetic variation and predisposition to cervical cancer only explain a small amount of the difference in underlying risk between individuals [1]. HrHPV is the most common sexually transmitted infection with over 70% of women being infected during their lifetime [2]. Most hrHPV infections are cleared by incompletely understood immune response, the epidemiological and lifestyle factors leading to hrHPV persistence, and especially neoplastic progression, are not fully understood.

Over the last three decades, many epidemiological studies have investigated the risk factors associated with hrHPV persistence and development of cervical cancer including immunosuppression [3–6], concomitant sexual infections [7–9], risky sexual behaviour [10, 11] and tobacco smoking [12]. However, most behavioural, environmental, or comorbid exposures are not suitable for investigation by randomised design trials and the evidence base is therefore subject to inherent biases. For some reported associations, a wide range in the magnitude of the effect size has been observed and studies have reported opposing directions of effect for the same exposure, such as for early age of first pregnancy [13, 14] or tobacco smoking [4, 15]. Determining the true strength of an association from p-values alone can be misleading, and selective reporting of positive results can also lead to an overestimation of the strength of an association. Previous umbrella reviews have demonstrated that despite numerous reported significant associations across differing scientific specialties, very few survive more rigorous analyses [16–21]. Although umbrella reviews offer a further appraisal of the evidence, they cannot infer causality if the underlying studies are observational in design. To explore the potential causal relationships of identified exposures, Mendelian randomisation (MR) can be useful and complementary to traditional observational studies, as genetic instruments, where available, can control for a degree of unknown confounding.

We performed an umbrella review of systematic reviews and meta-analyses exploring the association between modifiable risk factors and environmental exposures and hrHPV infection, cervical intraepithelial neoplasia (CIN) and cervical cancer incidence and mortality. We further conducted a MR analysis, to assess the strength and validity and potential causal effect of previously reported estimates.

## Methods

### Search strategy and selection criteria

**Fig. 1:**
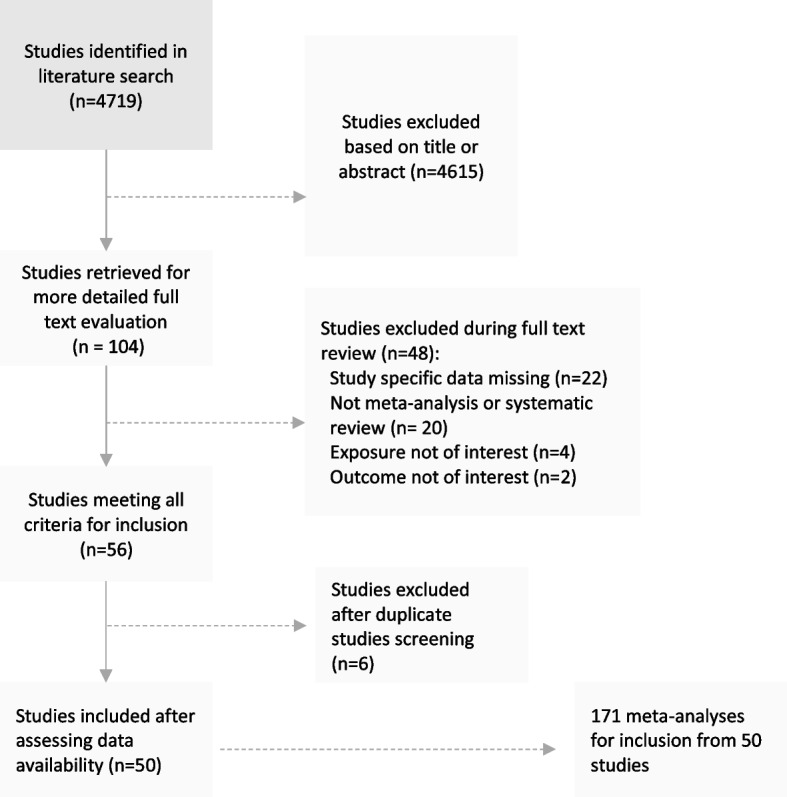
PRISMA flow chart of study-selection

We searched PubMed, Ovid MEDLINE and Embase Classic, and the Cochrane database for systematic reviews to investigate the association between non-genetic behavioural, environmental, or comorbid risk factors and incidence or prevalence of hrHPV, CIN or cervical cancer, cervical cancer mortality, and regression or progression of disease. All articles were screened at least in duplicate (SB, TD and AA) using pre-defined search terms (Additional file 1 - Supplementary Methods). We further hand-searched the reference lists of included papers for and the proceedings of relevant conferences for unpublished data (SB, TD and AA) (Fig. 1). The protocol for this study is available on PROSPERO (CRD42020189995). We included all systematic reviews and meta-analyses of observational or interventional studies on behavioural, environmental or co-morbid risk factors affecting hrHPV, CIN or cervical cancer incidence or mortality - including outcomes concerning disease regression and progression. We excluded studies investigating genetic risk factors. We also excluded meta-analyses that did not report the necessary study-specific data including the relative risk (RR), 95% confidence intervals (CI), the number of cases/controls or total population (where we could not retrieve data from original studies). Where more than one meta-analysis examined the same exposure-outcome pair, we chose the meta-analysis containing the largest number of cohort studies. If the same number of cohort studies were included, we included the more recently published meta-analysis.

### Data extraction

We extracted the individual study level data for each study within each meta-analysis including the number of cases and controls or total population and the maximally adjusted relative risk (further details provided in Additional file 1 - Supplementary Methods). All the data extraction was performed at least in duplicate (TD, AA, SB and HGS), with any discrepancies resolved by discussion with a third investigator (IK).

### Data analysis and evaluating the strength of evidence by grading criteria

For each exposure and outcome pair, we calculated the summary effect and the 95% CI using fixed and random effects methods [22]. The heterogeneity between studies was assessed with Cochran’s Q test [23] and the *I*^2^ statistic [24] with 95% CI [25]. To further account for heterogeneity between studies we calculated 95% prediction intervals [26, 27] for the summary random effect estimates. We assessed whether smaller studies gave higher risk estimates than larger studies - an indication of publication bias, true heterogeneity, or chance. To assess small study effects, we used Egger’s regression asymmetry test (*P* ≤ 0.10) [28] and whether random effects summary estimates are larger than the point estimate of the largest study in the meta-analysis. We assessed excess significance bias by evaluating whether the observed number of studies with nominally statistically significant results (*p* < 0.05) in the published literature is different from the expected number of studies [25]. Finally, we used a credibility ceilings threshold to account that a single observational study cannot give more than a maximum certainty [29]. We graded the strength of evidence into strong, highly suggestive, suggestive and weak using criteria as previously described [16, 17] and are outlined in the supplementary material (Additional file 2 - Supplementary Table 1).

### Evaluation of the quality of included meta-analyses

We used the AMSTAR-2 criteria [30] to assess the quality of the evidence. Evidence was graded based on the strength and validity as according to previous published umbrella reviews [16, 17]. The four grades range from ‘high’ to ‘critically low’, with ‘high’ evidence having zero or one non-critical weakness in the study, and ‘critically low’ having more than one critical flaw, with or without a non-critical weakness (Additional file 1 - Supplementary Methods).

### Sensitivity analyses

The main analysis was restricted to cohort studies only. We conducted a sensitivity analysis which included all eligible observational studies, i.e. meta-analyses including both cohort and case–control studies. These were then graded using the same criteria as for the main analyses.

In the event of multiple meta-analyses reporting on the same exposure-outcome associations, we selected the one with the largest number of studies to prevent duplication of the original studies. The concordance between the included and duplicate meta-analyses was explored in a sensitivity analysis (Additional file 1 - Supplementary Methods).

### Mendelian randomisation

To investigate the potential causal effects of proposed environmental risk factors on cervical cancer, we conducted a two-sample MR analysis, which uses genetic variants with known effects on the risk factor, as a proxy for the exposure [31, 32].

For all exposures graded as weak evidence or above in the umbrella review, we searched the GWAS Catalog [33] to identify relevant GWAS studies providing female-specific summary-level genetic data. We used data from a previously published genome-wide association study to obtain associations of SNPs with risks of cervical cancer [1].

We used inverse variance weighted (IVW) MR as the main analysis to estimate the causal effect on risk for cervical cancer [34]. For exposures with significant effects in the main IVW MR analysis (multiple testing adjusted using Benjamini and Hochberg’s false discovery rate [35]), we performed sensitivity analyses using a range of robust MR approaches to circumvent possible violations of the instrumental variable assumptions (weighted median [36], MR-Egger [37], and Mendelian Randomisation Pleiotropy RESidual Sum and Outlier (MR-PRESSO) [38] (Additional file 1 - Supplementary Methods)).

### Role of the funding source

The funder of the study had no role in study design, data collection, data analysis, data interpretation, or writing of the report.

## Results

The literature search was performed in November 2020 and identified 4722 systematic reviews or meta-analytical papers. Following sequential title, abstract and full-text screening, 56 meta-analysis papers met the inclusion criteria. After exclusion of duplicate meta-analyses, 50 papers remained [4, 7, 10, 12, 14, 39–83], which included 171 separate meta-analyses of 1513 individual primary studies, (447 were cohort studies and 1056 case–control) (Fig. 1); no randomised controlled trials were identified.

### Characteristics of the meta-analyses

A total of 11 outcomes were identified, which related to either HPV infection, CIN and/or cervical cancer: cervical disease regression (hrHPV clearance; CIN regression), disease incidence and prevalence (hrHPV incidence; hrHPV prevalence; CIN incidence; CIN prevalence; cancer incidence), disease persistence (hrHPV persistence; CIN persistence), disease progression (CIN progression) and cancer mortality.

Fifty exposures were identified, which belonged to eight broad categories: immunocompromise (HIV infection, inflammatory bowel disease (IBD) on medication, rheumatoid arthritis (RA), systemic lupus erythematous (SLE), transplant recipients); co-infection and vaginal microbiome (VMB), (Epstein-Barr virus (EBV) infection, *Chlamydia trachomatis*, *Trichomonas*, *U. urealyticum,* Mycoplasmas, *Candida albicans*, vaginal *Lactobacillus* spp., herpes simplex virus (HSV) infection); anthropometric measures (body mass index (BMI), height); lifestyle and/or behavioural factors (smoking, alcohol intake, number of sexual partners); medical co-morbidities and/or medication use (retinoid use); gynaecological and obstetric factors (parity, age of first pregnancy, pregnancy, in vitro fertilisation (IVF), gestational diabetes mellitus (GDM), vaginal douching); hormonal medication use (combined oral contraceptive pill (COCP), intrauterine devices (IUD), injectable contraception); dietary, vitamin or antioxidant intake (vitamins A, E, C, lycopene, folate and carotenoids, vegetable/fruit, zinc, copper and selenium).

### Quality assessment

Twelve per cent of all the included papers were graded as high quality (1/50, 2%) [54] or moderate quality (5/50, 10%) [7, 41, 42, 60, 63], with Helm et al. [54] being the only research study that adequately fulfilled all the major components of the AMSTAR2 questionnaire. On the contrary, 14% (7/50) and 74% (37/50) of meta-analytical papers were graded as low or critically low quality, respectively (Additional file 3 - Supplementary Table 2). Papers characterised as ‘low’ or ‘critically low’ quality failed to meet criteria related to protocol, literature search, description of excluded studies and risk of bias assessment. Specifically, only 18% (9/50) listed the excluded studies and reason for exclusion. Additionally, 38% (19/50) did not use a pre-registered protocol, while 20% (10/50) did not report screening the literature adequately (either because they searched only one electronic database and/or did not provide a search strategy). Study selection and data extraction were performed in duplicate in 40% (20/50) and 42% (21/50), respectively. Most provided satisfactory methods for the risk of bias assessment (80%, 40/50), statistical analysis (76%, 38/50), interpretation of the main results (70%, 35/50) and investigations of small study effects (66%, 33/50).

### Main analysis

**Fig. 2:**
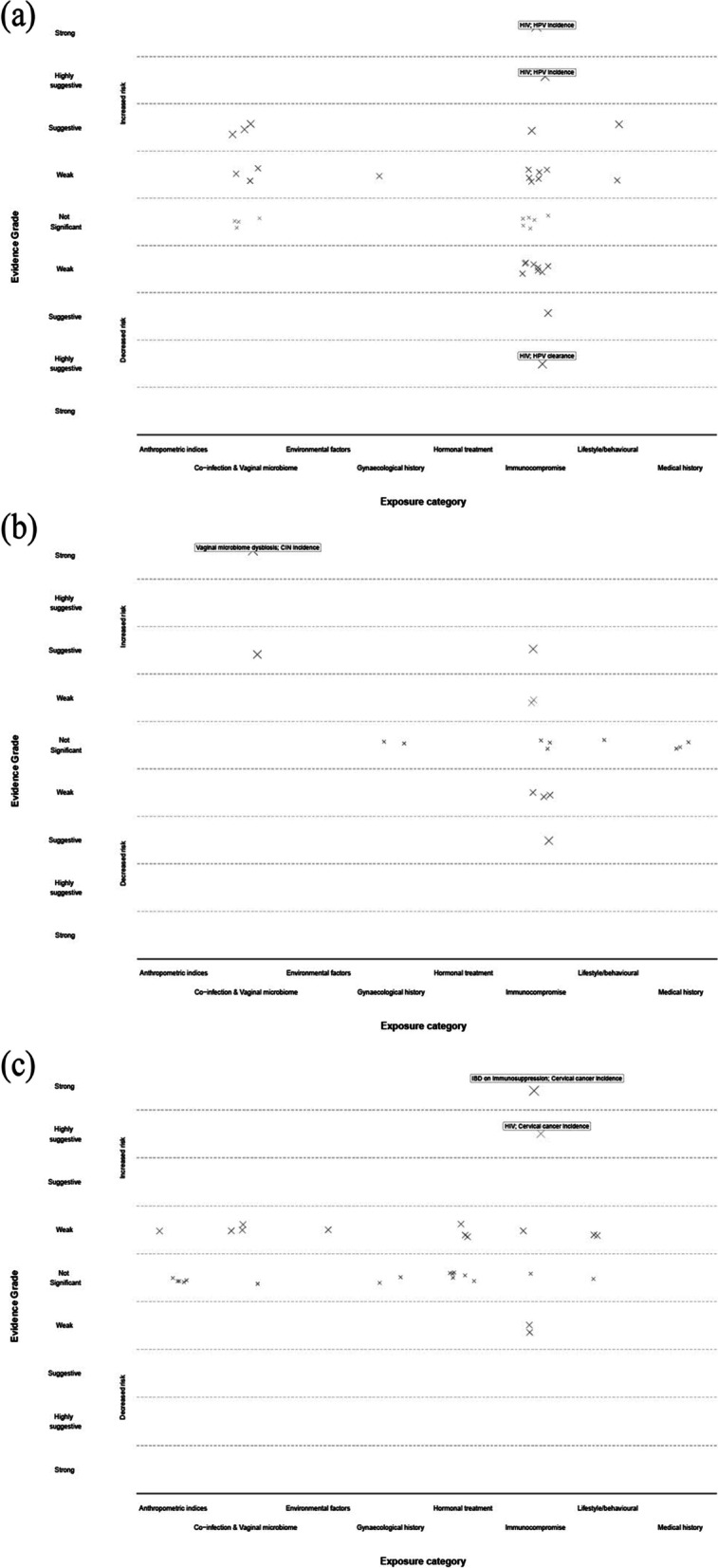
Distribution of studies across evidence grade for all exposures of either increased or decreased risk (*y*-axis) by exposure category (*X*-axis) from the main analysis (summary random effects for cohort studies only) and outcome **a** HPV infection; **b** cervical intraepithelial neoplasia; and **c** cervical cancer

Of the 171 meta-analyses, 87 included two or more cohort studies and were included in the main analysis and assessed a total of 39 exposures across the eight categories (Table 1, Fig. 2).

**Table 1:** Summary of evidence grading for meta-analysis of risk factors associated with HPV infection and pre-invasive and invasive cervical cancer outcomes — cohort studies only^a^

Evidence	HPV-related outcomes	CIN and cervical cancer-related outcomes
	**Increased risk**	**Increased risk**
**Strong**	HIV: positive vs negative (HR-HPV incidence)	IBD on immunosuppression vs healthy controls (CC incidence); VMB: dysbiosis vs no (progression to dysplasia and CIN)
**Highly suggestive**	HIV: positive with CD4 > 200 vs negative (HPV incidence) HIV: positive vs negative (HPV clearance)^b^	HIV: positive vs negative (CC incidence)
**Suggestive**	HIV: positive vs negative (HPV incidence); HIV: positive vs negative (HR-HPV persistence) VMB: LL vs HL (HPV incidence); VMB: dysbiosis vs no (HPV incidence); Chlamydia tr: yes vs no (HPV incidence); smoking: yes vs no (HPV incidence)	HIV positive: on ART vs not on ART (CIN regression); HIV: positive vs negative (CIN persistence); bacterial vaginosis: yes vs no (CIN prevalence)
**Weak**	Smoking: yes vs no (HPV incidence); VMB: dysbiosis vs no (HPV persistence); bacterial vaginosis: yes vs no (HPV incidence); Chlamydia tr: yes vs no (HR HPV incidence); pregnant: yes vs no (HPV incidence); HIV: positive vs negative (HPV incidence, HR HPV incidence, HPV 16 incidence, HPV 18 incidence); HIV: positive with CD4 < / = 200 vs negative (HPV incidence); HIV: positive with CD4 > 200 vs negative (HR-HPV incidence); HIV: positive vs negative (prevalent and newly detected HR-HPV, HPV 16, HPV 18, HPV-any type persistence); HIV: positive vs negative (HPV 16 persistence); HIV + ve: CD4 < 200 vs CD4 > 500 (HPV persistence); HIV + ve: CD4 200–500 vs CD4 > 500 (HPV persistence)	COCP: < 5 years of use vs never (ICC incidence); COCP: 5–9 years of use vs never (ICC incidence); COCP: > 10 years of use vs never (ICC incidence); smoking: current smoker vs never (CC incidence); smoking: previous smoker vs never (CC incidence); environmental tobacco smoke exposure: increased vs lower (CC incidence); HIV: positive vs negative (CIN incidence, CIN persistence); HIV positive: on ART vs not on ART (CIN incidence^b^, CIN progression^b^, ICC incidence^b^); transplant recipient: yes vs no (CC incidence); rheumatoid arthritis: yes vs no (CC incidence); BMI: highest vs lowest levels (CC mortality); Chlamydia tr: yes vs no (CC incidence); co-infection of Chlamydia tr and HPV: yes vs no (CC incidence)

*Abbreviations*: *ART* Antiretroviral treatment, *BMI* Body mass index, *BV* Bacterial vaginosis, *CC* Cervical cancer, *Chlamydia tr* Chlamydia trachomatis, *CIN* Cervical intraepithelial disease, *COCP* Combined oral contraceptive pill, *HIV* Human immunoinsufficiency virus, *HL* High in lactobacillus, *HPV* Human papillomavirus, *HR-HPV* High-risk HPV, *IBD* Inflammatory bowel disease, *ICC* Invasive cervical cancer, *LL* Low in lactobacillus, *VMB* Vaginal microbiome

^a^Only meta-analyses meeting at least a weak grade of evidence listed

^b^Decreased risk

#### Summary effect size

Applying *p* < 0.05 as a threshold for the level of significance, the summary fixed effects estimates were significant in 62% of cohort studies (54/87) and the summary random effects in 60% (52/87) of the meta-analyses (Additional file 4 - Supplementary Table 3). When using *p* < 0.001 as a cut-off, 53% (45/87) and 39% (34/87) of the studies presented significant summary fixed and random effects estimates, respectively. Where a cut-off of *p* < 10^−6^ was applied, 32% (28/87) and 19% (16/87) of the meta-analyses produced significant summary results in the fixed and random effects model, respectively. Out of the 16 meta-analyses with a random *p* < 10^−6^, 14 exposures were associated with an increased risk of either hrHPV incidence or persistence, or the increased risk of progression to LSIL or HSIL, CIN or cervical cancer (vaginal dysbiosis, HIV + , IBD on immunosuppression, *Chlamydia trachomatis* infection and co-infection with hrHPV, smoking and rheumatoid arthritis), while two exposures were associated with a decreased risk for hrHPV clearance (HIV + ; HIV + with low CD4 + cell count).

#### Heterogeneity between studies

The Cochrane’s Q test for heterogeneity was significant at *p* ≤ 0.10 in 27 of 87 meta-analyses (31%). Twenty-five studies (29%) presented a high heterogeneity (*I*^2^ = 50–75%) and nine (10%) very high (*I*^2^ > 75%), which included 5 different exposures (HIV + ; HIV + on treatment; bacterial vaginosis; *Chlamydia trachomatis*; pregnancy). When calculating the 95% prediction intervals, the null hypothesis was excluded for nine associations (IBD on immunosuppression – cervical cancer incidence; vaginal dysbiosis – hrHPV incidence; vaginal dysbiosis – progression from normal to LSIL or HSIL; HIV + – hrHPV incidence; HIV + – CIN2 + treatment failure; HIV + – CIN1 + treatment failure; smoking – hrHPV incidence; smoking – cervical cancer incidence; COCP – cervical cancer incidence) (Additional file 5 - Supplementary Table 4).

#### Small study effects

In three meta-analyses, there was evidence of small study effects (Egger’s test of *P* < 0.10 and detection of more conservative effects in the largest study of a meta-analysis compared with the summary random effects estimate): *Chlamydia trachomatis* infection – hrHPV incidence, HIV + – CIN1 + treatment failure, and gestational diabetes mellitus – cervical cancer incidence (Additional file 5 - Supplementary Table 4).

#### Excess significance

When using the largest study estimate as the plausible effect size, evidence of excess significance was observed in eight meta-analyses (9%) of varying exposures including bacterial vaginosis, *Chlamydia trachomatis* infection, HIV positivity, RA, BMI, and current pregnancy. Using the fixed or random effect estimate as the plausible effect sizes, five and four meta-analyses presented excess significance bias respectively (Additional file 5 - Supplementary Table 4).

#### Grading the evidence

**Fig. 3:**
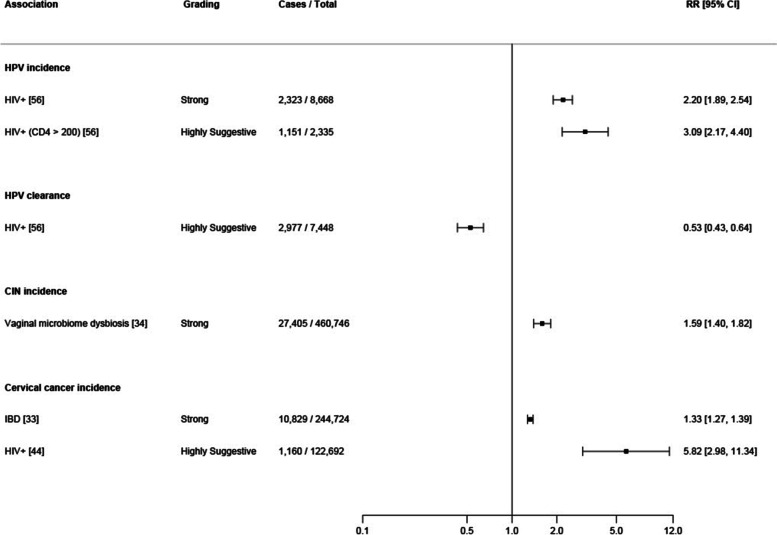
Forest plot of effect estimates and 95% confidence intervals for all exposure-outcome pairs for all outcomes (HPV, CIN (cervical intraepithelial neoplasia) and cervical cancer) in the main analysis (summary random effects for cohort studies only), which graded as strong or highly suggestive evidence (*n* = 6)

Of the 87 meta-analyses included in the main analysis, three met criteria to be graded as strong evidence, a further three as highly suggestive, nine as suggestive, while thirty-seven meta-analyses were of weak evidence; the remaining meta-analyses showed null associations (Additional file 6 - Supplementary Table 5). Out of the meta-analyses with strong and highly suggestive evidence, an increased risk of hrHPV and HPV incidence was associated with HIV positivity (strong evidence, *N* = 2323, RR 2.20, 95% CI 1.89–2.54, *p* = 3.01 × 10^−26^, *I*^2^ = 22 and highly suggestive evidence, *N* = 1151, RR 3.1, 95% CI = 2.17–4.4, random *p* = 3.75 × 10^−10^, *I*^2^ = 82, respectively), while a decreased risk of hrHPV clearance was also associated with HIV positivity (highly suggestive evidence, *N* = 2977, RR 0.53, 95% CI = 0.43–0.65, *p* = 4.32 × 10^−10^, *I*^2^ = 73). An increased risk for progression from normal to LSIL or HSIL was related to vaginal dysbiosis (strong evidence, *N* = 27,405, RR = 1.6, 95% CI = 1.4–1.81, *p* = 5.34 × 10^−12^, *I*^2^ = 26) and an increased risk for cervical cancer incidence was associated with people with IBD using immunosuppressive medications (strong evidence, *N* = 10,829, RR = 1.33, 95% CI = 1.89–2.54), *p* = 7.78 × 10^−37^, *I*^2^ = 0) and with HIV positivity (highly suggestive evidence, *N* = 1160, RR = 5.82, 95% CI = 2.98–11.34, *p* = 2.34 × 10^−07^, *I*^2^ = 86) (Fig. 3).

Two of the meta-analyses [41, 42] that met criteria for strong evidence were of moderate quality on AMSTAR assessment while the third [64] was evaluated as critically low quality. The most common reason for lower quality on AMSTAR for those studies was no report of the funding source, no description of the excluded studies, partial description of the included studies and for the search strategy. Similarly, most of the meta-analyses that were graded as highly suggestive evidence were graded as critically-low quality on AMSTAR assessment for the above reasons as well as for poor study design.

### Sensitivity analyses

Of the 87 meta-analyses in the main analysis, 37 (43%) retained nominal statistical significance (*P* < 0.05) with a credibility ceiling of 5%. With ceilings of 10%, 15% and 20%, twenty-two (25%), fourteen (16%) and four (5%) studies remained significant respectively (Additional file 7 - Supplementary Table 6).

When also including case–control studies, 11/171 meta-analyses met the criteria for strong evidence, including eight additional exposure-outcome pairs (Additional file 8 - Supplementary Table 7). Increased risk of hrHPV incidence was associated with *Chlamydia trachomatis* infection (*N* = 5049, RR = 2.32, 95% CI = 2.02–2.65, *p* = 3.45 × 10^−34^, *I*^2^ = 0%), while increased risk of CIN was related to multiple sexual partners CIN (*N* = 5638, RR = 1.97, 95% CI = 1.8–2.15, *p* =  < 1 × 10^−100^, *I*^2^ = 0%). An increased risk of cervical cancer incidence was associated with not only *Chlamydia trachomatis* infection (*N* = 3392, RR = 2.19, 95% CI = 1.74–2.74, *p* = 1.03 × 10^−11^, *I*^2^ = 47%), but also with *Trichomonas Vaginalis* infection (*N* = 7715, RR = 2.09, 95% CI = 1.69–2.6, *p* = 2.33 × 10^−11^, *I*^2^ = 34%) and oral contraception use (*N* = 5839, RR = 2.13, 95% CI = 1.87–2.42, *p* = 4.37 × 10^−31^, *I*^2^ = 1.4%). Meanwhile, co-infection of hrHPV with *Chlamydia Trachomatis* seems to increase the risk of cervical cancer by more than four times (*N* = 1086, RR = 4.37, 95% CI = 2.75–6.96, *p* = 4.59 × 10^−10^, *I*^2^ = 44%), while in a subgroup analysis *Chlamydia trachomatis* infection appears to increase the risk of squamous cell cervical cancer incidence (*N* = 3198, RR = 2.09, 95% CI = 1.79–2.44, *p* = 6.87 × 10^−21^, *I*^2^ = 32%) (evidence for adenocarcinoma was weak only).

Of the eight additional associations that met strong criteria in the sensitivity analysis, five were graded as weak or did not present nominally statistically significant association at *p* < 0.05 when only cohort studies were included. The remaining three included only case–control studies and were not assessed in the main analysis. All three strong associations from the main analysis remained strong when case–control studies were included. 14 studies met criteria for highly suggestive evidence, 27 met criteria for suggestive and 69 studies were classified as weak evidence only; the remaining 51 meta-analyses did not meet criteria for weak evidence (Additional file 8 - Supplementary Table 7).

We identified duplicate meta-analyses meeting inclusion criteria for six exposure-outcome pairs. For all duplicates the same direction of effect was observed in both the magnitude and significance of the summary associations, between included and excluded studies (Additional file 9 - Supplementary Table 8).

### Mendelian randomisation

In the MR analysis, 11 exposures had genetic instruments available to perform MR (Additional file 10 - Supplementary Table 9), namely smoking using a lifetime smoking index (which is a composite score that captures the lifetime smoking exposure by taking into account smoking status, as well as smoking duration, heaviness and cessation) [84], reproductive behaviour in women as measured by age at first pregnancy [85], number of sexual partners [86], lupus [87], rheumatoid arthritis, IBD, alcohol consumption [88], BMI, GDM, parity (number of living births) and height [89]. No hrHPV infection phenotypes were identified; however, genetic instruments for the aggregate CIN3 and cervical cancer phenotypes were available. The characteristics of the GWAS studies from which we selected the genetic instruments can be found in the supplement (Additional file 11 - Supplementary Table 10) and in the originally published studies.

**Fig. 4:**
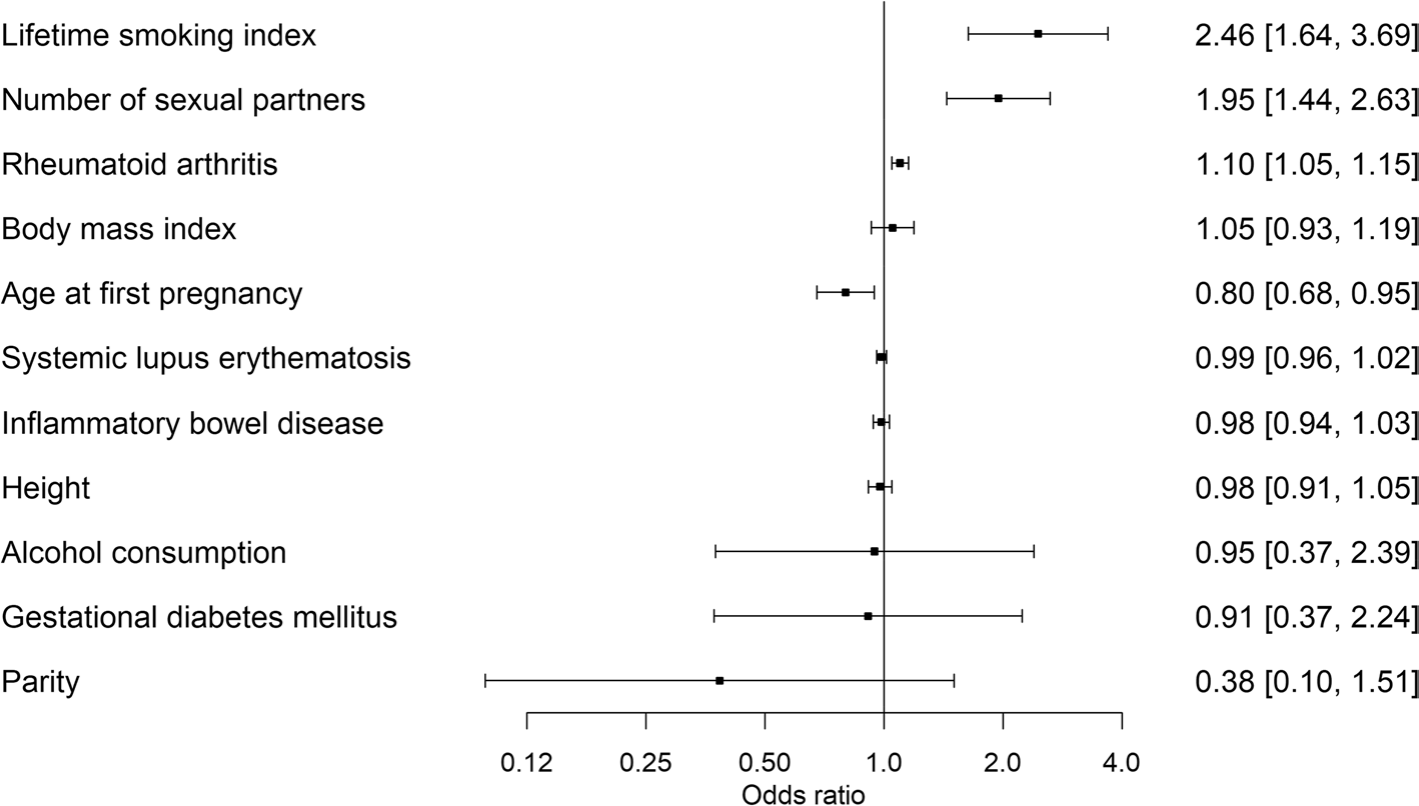
Forest plot demonstrating inverse variance weighted Mendelian randomisation results for all identified known environmental risk or protective factors for cervical cancer with available GWAS, to determine effect sizes by OR and 95% CI (*x*-axis; *n* = 11)

**Fig. 5:**
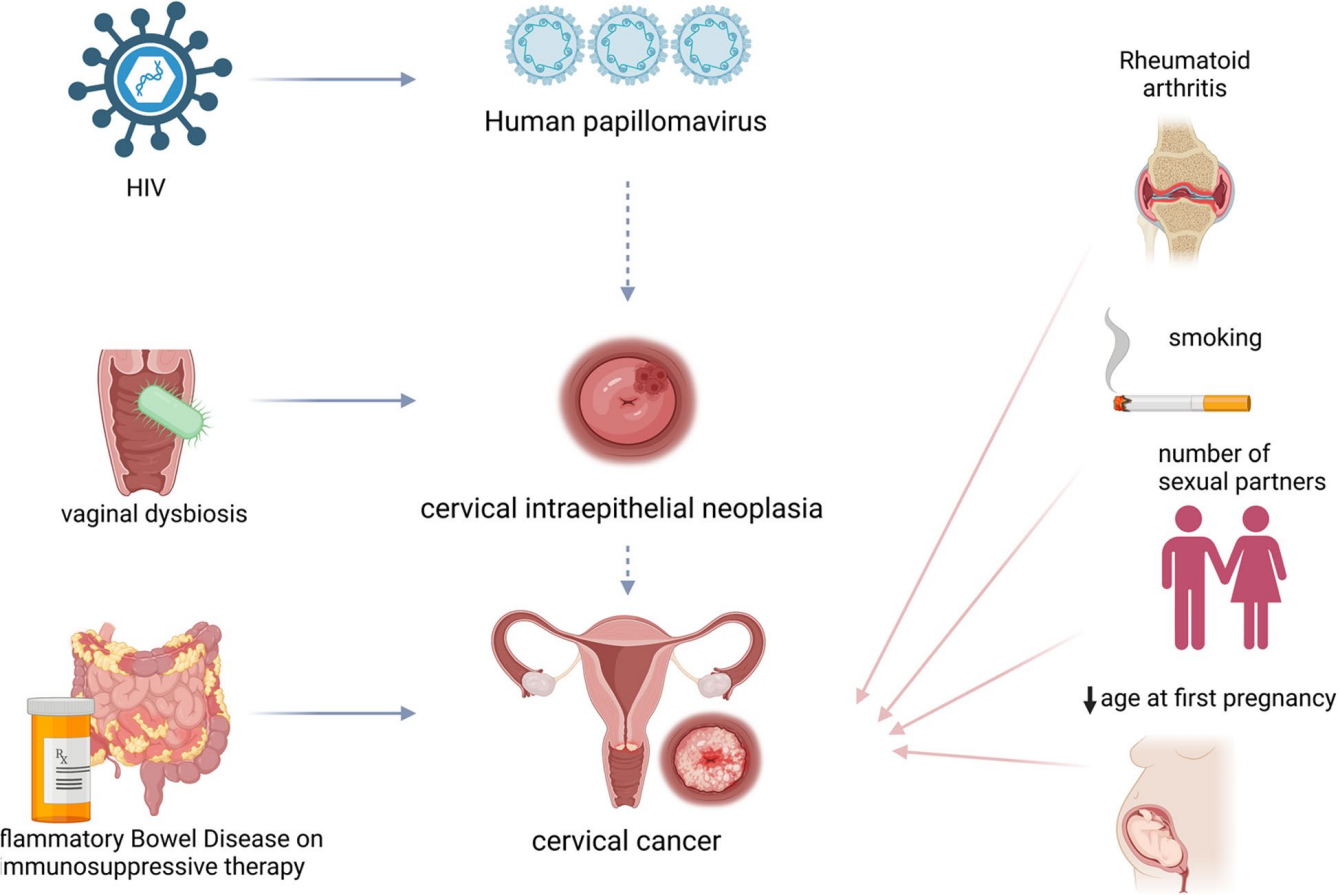
Summary of results from umbrella review and Mendelian randomisation: associations with strong evidence in the main analysis (left): HIV for human papillomavirus incidence, vaginal dysbiosis for development of cervical intraepithelial neoplasia, and inflammatory bowel disease on immunosuppressive therapy for cervical cancer and Mendelian randomisation results supporting an association with cervical cancer incidence (right: rheumatoid arthritis, smoking, number of sexual partners and older age at first pregnancy (protective effect))

The strongest associations with risk of cervical cancer were observed for genetically predicted lifetime smoking index (OR = 2.46, 95% CI = 1.64–3.69) and number of sexual partners (OR = 1.95, 95% CI = 1.44–2.63) (Fig. 4 and Additional file 12 - Supplementary Table 11). We additionally identified a protective effect for older age at first pregnancy (OR = 0.80, 95% CI = 0.68–0.95), while genetically predicted liability to rheumatoid arthritis increased the risk of cervical cancer (OR = 1.10 95% CI = 1.05–1.15) (Fig. 5). The associations of genetically predicted lifetime smoking index, age at first pregnancy, liability to rheumatoid arthritis, and number of sexual partners, with cervical cancer risk were supported in sensitivity analysis (Additional file 13 - Supplementary Tables 12–13). Genetically predicted age of first pregnancy, and lifetime smoking index, were independently associated with cervical cancer when controlling for genetically predicted number of sexual partners. Genetically predicted liability to SLE, IBD, GDM, and genetically predicted alcohol consumption, BMI, nulliparity and height all showed no significant association with CIN3 or cervical cancer (Additional file 13 - Supplementary Tables 12–13).

## Discussion

We present an umbrella review of 87 meta-analyses of observational studies on life course exposures including co-morbidities, environmental and behavioural exposures and risk of HPV infection, CIN and invasive cervical cancer (ICC). HIV, vaginal dysbiosis (*Lactobacillus* spp. depletion) and immunosuppressive medications in women with IBD were all supported by strong evidence, while smoking, Chlamydia infection and bacterial vaginosis were supported by highly suggestive or suggestive evidence. Sensitivity analyses also identified strong or highly suggestive evidence for an association between *Trichomonas* infection, increased number of sexual partners, medium- to long-term COCP usage, high parity, earlier age at first pregnancy, low vegetable intake, increased vitamin C or selenium intake, and HPV, CIN, or ICC.

We identified meta-analyses with strong, highly suggestive, and suggestive evidence for an increased risk of hrHPV and cervical cancer incidence in HIV-positive women, when compared to the general population. There was also highly suggestive evidence that HIV positivity reduces the risk of hrHPV clearance, whereas suggestive evidence exists that antiretroviral treatment (ART) increases CIN regression rates. The included studies were graded as low and critically low in the AMSTAR assessment while there were no genetic instruments to perform an MR analysis. Our findings support that of a recent study published jointly by the WHO and the International Agency for Research on Cancer (IARC), which reported that women living with HIV have a six-fold higher risk of developing cervical cancer [6]. Immunosuppression is considered the primary mechanism of many HIV-related diseases, and of HPV persistence [90], while genetic studies of women with CIN3 and cervical cancer have identified more frequent mutations in the HLA region coding for MHC class 2 cell production and T-cell activation [1]. Early ART initiation is thought to improve HPV clearance and CIN regression through maintenance of high levels of CD4 + T-cells [58].

The evidence for women with other forms of chronic immunosuppression and cervical cancer was more limited. We found strong evidence in the main analysis that women with IBD on immunosuppression are at increased risk for high-grade CIN and cervical cancer compared to the general population. The quality of the study scored moderate on AMSTAR while MR analysis didn’t show any significant association. It is unclear, though, whether IBD alone is associated with cervical cancer regardless of medication.

We found weak evidence for an increased risk of cervical cancer for other autoimmune diseases, including a meta-analysis on SLE and CIN. Multiple studies have suggested that SLE alone is associated with hrHPV acquisition [91], CIN [91–96] and that the use of immunosuppressants in SLE patients is associated with an increased incidence of cervical dysplasia [96, 97]. The risk associated with RA was also weak. People living with rheumatoid arthritis have been found to be at significantly higher risk of other cancers including lymphoma and lung cancer, compared to the general population [70, 98]. As well as the immune dysfunction induced by RA itself, it is likely that immunosuppressive medications like steroids and disease-modifying antirheumatic drugs (DMARDS) which can decrease the number and function of T-cells [99] play a role in the risk of cancer. Although MR did not support a causal association for genetically predicted liability to IBD and SLE to CIN3 and cervical cancer, there was nominal evidence of causality for genetically predicted liability to RA (OR = 1.1, 95% CI = 1.05–1.15), it was not plausible however to assess the role of the disease alone in separation to immunosuppressive medications however. The studies for both SLE and RA were graded as critically low in the AMSTAR assessment mainly due to the quality of literature search, description of the included studies, funding, and statistical methods for the MA.

We found strong and suggestive evidence that a *Lactobacillus* spp. deplete VMB, as well as cervicovaginal infection (including *Chlamydia trachomatis* and *Trichomonas* spp.) increase the risk of hrHPV acquisition, CIN incidence and cancer incidence, including progression from normal cytology to HSIL. Evidence was particularly strong for progression to LSIL or HSIL [42] due to the large number of individuals from cohort studies (*N* = 460,746). The evidence of the association between chlamydia and hrHPV incidence [67] was downgraded to suggestive in the main analysis. The highest score in the AMSTAR assessment was moderate and that was for the study with strong evidence for altered VMB and CIN. The rest of the studies were graded as low or critically low. *Lactobacillus* spp. maintain a low pH and the acidic environment is essential for the function of the cervical epithelial barrier [100–102] and Chlamydia infection can disrupt the cervical epithelium, allowing increased HPV entry to basal epithelium [103–105]. Studies have demonstrated that VMB diversity increases with advancing CIN disease severity [106], while lactobacillus-depleted VMB is associated with a significantly lower chance of regression of untreated CIN1 when compared to Lactobacillus-dominant VMB [107]. There were insufficient genetic instruments to assess the VMB or vaginal infection with Chlamydia or Trichomonas with MR.

Although smoking is widely considered to be a strong risk factor for cervical cancer, the evidence was graded as suggestive between smoking and increased HPV incidence and as only weak between active smoking and ICC. The meta-analyses of ICC were considered to present only weak evidence of association due to the small number of cases, relatively large *p*-values and the presence of small study effects. All the studies investigating smoking scored critically low in AMSTAR, mainly due to study design, risk of bias assessment, heterogeneity and small study bias. Cigarette smoking is widely accepted as a strong carcinogen which hampers cellular immunity at the cervix [108] and this analysis highlights the inherent issues that can arise from the quality of observational research. Particularly, that the study of a rare outcome such as cervical cancer can be difficult in a cohort design and that larger numbers of cases can only be achieved in a case–control study. MR suggests a true causal effect that may have been obscured in meta-analyses based on cohort studies. For cigarette smoking, our MR suggested a strong causal effect of lifetime smoking index on an increased risk of cervical cancer (OR = 2.46, 95% CI = 1.64–3.69), this was still apparent when controlling for other risky behaviours.

There were no meta-analyses based on cohort studies for the evaluation of the sexual and reproductive history on cervical cancer. The association of multiple sexual partners [10, 70] with CIN and cervical cancer was supported with strong and highly suggestive evidence in a sensitivity analysis of 6 meta-analyses, and confirmed in the MR analysis, although it is challenging to instrument such a variable, which might largely reflect a general propensity for risky behaviours. The quality of the studies was low in AMSTAR. While an increased number of sexual partners is thought to increase hrHPV exposure, particularly at an early age, the mechanism regarding parity and early age of pregnancy is less well understood. It is possible that an early age of first pregnancy affects the transformation zone, increasing its vulnerability to infection [109, 110] or the immunosuppressed pregnant state increases vulnerability. However, high parity and early age of first pregnancy may be surrogate markers for increased HPV exposure at an earlier age. We explored the independence of age of first pregnancy and sexual behaviour via a multivariate MR. We observed that a genetically predicted young age of first pregnancy was independently associated with cervical cancer when controlling for risky behaviours such as genetically predicted higher number of sexual partners.

While BMI was linked to increased cervical cancer mortality possibly due to other obesity-related complications, there was no link to any other HPV or cervical cancer outcome, which was confirmed on MR. Our study provided only weak evidence that the use of COCP increases the risk of invasive cervical cancer, although the evidence became strong for medium to long-term COCP use when case–control studies were included as well [109].

We used a well-established methodology for this analysis [16, 17, 21, 111]. A lack of evidence does not infer the absence of an association; however, where a weak evidence grade was assigned, this may suggest the need for further good-quality studies. This is particularly true in the context of associations that are widely thought to be causal, as downgrading of evidence results from presence of biases in the evaluated literature, not from suspected absence of association. As causality cannot be inferred from observational research, and a lack of randomised research was observed from this systematic review, we performed an MR wherever genetic instruments were available. This is the first MR study in the field of cervical cancer risk and brings new insights to the possible causality from examined exposures.

Possible limitations should be considered in the interpretation of our findings. This review relies on the previously published meta-analyses and literatures searches performed by the authors of those studies. Some literature may have been missed; however, the assessment of duplicate analyses did not highlight any discordant results which minimises this risk. Additionally, we studied exposures against several outcomes linked to the development of cervical cancer, these results were also consistent across meta-analyses. Although the overall number of studies included was large, for some associations, the number of studies and participants was small, limiting our ability to assess for the presence of small study effects and excess significance due to low power. It is likely that this would result in a more conservative estimate and the true association may be more severe. Additionally, most studies in the umbrella were graded as low or critically low according to the AMSTAR2 criteria, suggesting a high risk of bias within this evidence. Although we assessed for risk of bias, the statistical tests are unable to explain the definitive presence or the likely source of bias. Furthermore, MR analyses were underpowered for some exposures (such as for IBD), and sample overlap in some of the associations was relatively large (such as the number of sexual partners) [112].

## Conclusions

There is consistently strong and highly suggestive evidence that HIV positivity reduces HPV clearance rates and increases the risk of HPV infection and cervical cancer development. Vaginal *Lactobacillus* spp. depletion and immunosuppressive medications for women with IBD are also strongly associated, with a suggestion that other forms of immunosuppression increase risk of cervical cancer. Our results suggest that the presence of *Chlamydia trachomatis* and *Trichomonas* infections influence the development of cervical cancer in the presence of hrHPV and prompt treatment should be prioritised. In conservatively managed HPV infections and CIN, additional screening and treatment for concomitant bacterial infection and bacterial vaginosis should be considered [113, 114]. This strengthens the call for more evidence on the role of probiotics in preventing HPV persistence and cervical cancer. While for cigarette smoking, we found highly suggestive evidence in case–control studies only, this is likely secondary to small numbers of cervical cancers and smoking cessation should still be recommended to women with cervical abnormalities. Young age of first pregnancy was independently associated with cervical cancer when controlling for other risky behaviours including a higher number of sexual partners and smoking.

This evidence highlights the importance of preventative strategies including the provision of sexual health and family planning services, with early initiation of ART in HIV-positive women, alongside cervical screening. The strong interaction between HIV and cervical cancer necessitates the prioritisation of HPV vaccination in populations where HIV prevalence is high in initiatives to increase global access.

## Supplementary Information

**Additional file 1: Supplementary Methods.** Additional details including electronic search terms and data analysis pipeline of Umbrella review.

**Additional file 2: Supplementary Table 1.** Statistical criteria for grading of the evidence.

**Additional file 3: Supplementary Table 2.** Summary quality assessment for all included systematic reviews using the ASMTAR 2 tool (A Measurement Tool to Assess Systematic Reviews 2).

**Additional file 4: Supplementary Table 3.** Description of 87 meta-analyses investigating risk factors associated with HPV, cervical pre cancer and cancer outcomes - only cohort studies included.

**Additional file 5: Supplementary Table 4.** Evaluation of heterogeneity, small study effects and excess significance bias in the 87 meta-analyses investigating the risk factors associated with HPV, cervical pre cancer and cancer outcomes - only cohort studies included.

**Additional file 6: Supplementary Table 5.** Details of evidence grading for meta-analysis of risk factors for HPV, cervical precancer and cancer outcomes – _only cohort studies included*.

**Additional file 7: Supplementary Table 6.** Sensitivity analysis using credibility ceilings when the association is non-significant of the 87 studies investigating the risk factors associated with HPV, cervical pre cancer and cancer outcomes - only cohort studies included.

**Additional file 8: Supplementary Table 7.** Details of evidence grading for meta-analyses of risk factors for HPV, pre-invasive and invasive cervical cancer incidence, progression, regression, or mortality - all study types included (sensitivity analysis).

**Additional file 9: Supplementary Table 8.** Excluded duplicate studies and studies selected in their place to be included in the analysis.

**Additional file 10: Supplementary Table 9.** Mendelian randomisation (MR) analysis; exposure-outcome pairs included in the main analysis, cohorts only.

**Additional file 11: Supplementary Table 10.** Main GWAS study characteristics.

**Additional file 12: Supplementary Table 11.** Two-sample inverse variance weighted mendelian randomization full results of the analyses of risk factors on cervical cancer.

**Additional file 13: Supplementary Table 12.** Results from Mendelian randomisation sensitivity analyses of risk factors on cervical cancer. **Supplementary Table 13.** Results from multivariable (MV) Mendelian randomisation (MR) sensitivity analyses of risk factors on cervical cancer.

## Data Availability

All data generated or analysed during this study are included in this published article [and its supplementary files].

## Abbreviations

AMSTAR: A MeaSurement Tool to Assess systematic Reviews
ART: Antiretroviral treatment
BMI: Body mass index
CD4: Cluster of differentiation 4
CI: Confidence interval
CIN: Cervical intraepithelial neoplasia
CIN1: Cervical intraepithelial neoplasia grade 1
CIN1 +: Cervical intraepithelial neoplasia grade 1 or higher
CIN2 +: Cervical intraepithelial neoplasia grade 2 or higher
CIN3: Cervical intraepithelial neoplasia grade 3
COCP: Combined oral contraceptive pill
DMARDS: Disease-modifying antirheumatic drugs
EBV: Epstein-Barr virus
GDM: Gestational diabetes mellitus
GWAS: Genome-wide association study
HIV: Human immunodeficiency virus
HLA: Human leukocyte antigens
HPV: Human papillomavirus
HrHPV: High-risk human papillomavirus
HSIL: High-grade squamous intraepithelial lesion
HSV: Herpes simplex virus
IARC: International agency for research on cancer
IBD: Inflammatory bowel disease
ICC: Invasive cervical cancer
IUD: Intrauterine devices
IVF: In vitro fertilisation
IVW: Inverse variance weighted
LSIL: Low-grade squamous intraepithelial lesion
MHC: Major histocompatibility complex
MR: Mendelian randomization
MR-PRESSO: Mendelian randomization - pleiotropy residual sum and outlier
OR: Odds ratio
PROSPERO: International prospective register of systematic reviews
RA: Rheumatoid arthritis
RR: Risk ratio
SLE: Systemic lupus erythematosus
SNPs: Single nucleotide polymorphisms
VMB: Vaginal microbiome
WHO: World Health Organisation
