# Supplementary material for: Risk factors for human papillomavirus infection, cervical intraepithelial neoplasia and cervical cancer: an umbrella review and follow-up Mendelian randomisation studies
Source: BMC Med. 2023 Jul 27;21:274. doi: 10.1186/s12916-023-02965-w (PMC10375747; doi:10.1186/s12916-023-02965-w)
Supplement: Supplementary file 1 — Additional file 1: Supplementary Methods. Additional details including electronic search terms and data analysis pipeline of Umbrella review. [file 12916_2023_2965_MOESM1_ESM.docx]

**SUPPLEMENTARY METHODS**

**Electronic search terms**

We searched the electronic databases PubMed, Ovid MEDLINE and Embase Classic, and the Cochrane database of systematic reviews for systematic reviews and meta-analyses of that investigated the association between non-genetic risk factors and incidence or prevalence of HPV, CIN or cervical cancer from inception to 1^st^ May 2021. The search strategy was developed with the Imperial College London (Hammersmith Hospital campus) librarian. The Pubmed search terms were as follows, the same search was adapted for the other databases:

(((((((Uterine Cervical Neoplasms [MeSH Terms] OR Uterine Cervical Dysplasia [MeSH Terms] OR female genital tract cancer OR uterine cervix cancer OR Cervical Intraepithelial Neoplasia [MeSH Terms] OR CIN [tw] OR CINII* [tw] OR CIN2* [tw] OR CINIII* [tw] OR CIN3* [tw] OR SIL [tw] OR HSIL [tw] OR H-SIL [tw] OR LSIL [tw] OR L-SIL [tw] OR ASCUS [tw] OR AS-CUS [tw]) OR (papillomaviridae [MeSH:NoExp] OR alphapapillomavirus [MeSH Terms] OR "DNA, viral" [MeSH Terms] OR Papillomavirus Infections [MeSH Terms] OR Tumor Virus Infections [MeSH Terms] OR "Cervix Uteri/virology" [MeSH Terms] OR HPV [tw] OR "human papillomavirus" [tw] OR papillomaviridae [tw]))) OR ((cervix[Tw] OR cervical[Tw] OR cervico*[Tw] OR Cerv*[tw]) AND (cancer*[Tw] OR carcinoma[Tw] OR adenocarcinoma[Tw] OR neoplas*[Tw] OR dysplas*[tw] OR dyskaryos*[Tw] OR squamous[Tw]))) AND (Meta-analysis[pt] OR systematic[sb] OR (Meta-analysis[tw] OR systematic review[tw] OR systematic[sb] OR meta-analysis[pt] OR meta-analysis as topic[mh] OR meta-analysis[mh] OR meta analy*[tw] OR metanaly*[tw] OR metaanaly*[tw] OR met analy*[tw] OR systematic review*[tiab])))) NOT (animal[mh] NOT human[mh]) Sort by: PublicationDate

**Data extraction**

We extracted the name of the first author and the year of publication, the modifiable exposure and outcome studied, and the summary effect (relative risk, odds risk, hazard ratio, standard incidence ratio, standard mortality ratio), and 95% confidence intervals (CI) or standard error (SE) from each eligible meta-analysis. From each individual study in a meta-analysis, we then extracted the first author and the publication year, epidemiological design (cohort, case control), number of cancer cases and controls in case- control studies or the number of cases and total population or person years in cohort studies, maximally adjusted relative risk (odds ratio in case-control studies, risk ratio or hazard ratio in cohort studies and the 95% confidence intervals. Meta-analyses were evaluated as they were originally presented; expansion of one meta-analysis with studies detected by another on the same topic was beyond the scope of this review.

**Data Analysis**

*Assessment of summary effect, heterogeneity and prediction intervals*

For each exposure, we calculated the summary effect and 95% confidence interval using both fixed and inverse variance weighted random effects methods. We used the Cochran Q test and the I^2^ metric of inconsistency including its 95% confidence intervals to assess for inter-study heterogeneity. The I^2^ metric ranges between 0% and 100% and could reflect either genuine diversity within the studies, chance or bias. A further assessment of inter-study heterogeneity was performed by calculating the 95% prediction intervals (PI) for the summary random effect estimates. The PI enables direct comparison with future clinically relevant effect estimates as it provides a range within which the effect estimate is predicted to fall.

*Assessment of small study effects*

To evaluate whether the smaller studies in a meta-analysis were creating an exaggerated risk estimate relative to the larger studies, we used Egger’s test at p<0.10 with the random-effects summary estimate being further away from the null-value compared to the point estimate of the largest study in a meta-analysis (i.e. the study with the smallest standard error). Small study effects can occur due to publication or reporting bias, true heterogeneity or chance.

*Evaluation of excess statistically significant bias*

The test for excess significance aims to evaluate whether the observed (O) number of statistically significant results (*p*<0.05) in the studies included in a meta-analysis is too large compared to the expected (E) number. The number of significant studies that were expected in each meta-analysis was calculated using the sum of the statistical power estimates for each included study using non-central *t*-distribution. The effect of the largest study (i.e. has the smallest standard error) was used as the plausible effect size as the true effect size is not known. Sensitivity analyses were performed using the summary fixed and random effect estimates as alternative plausible effect sizes. Excess significance for each individual meta-analysis was defined as two-sided *P*<0.10.

*Evaluating the strength of the evidence by grading*

The expected number of statistically significant studies in each meta-analysis is calculated from the sum of the statistical power estimates for each component study using an algorithm from a non-central *t* distribution. The power estimates of each component study depend on the plausible effect size for the tested association, which is assumed to be the effect of the largest study (that is, the smallest standard error) in each meta-analysis. Excess significance for individual meta-analyses is determined at P≤0.10.

We used credibility ceilings, a sensitivity analysis tool, to account for potential methodological limitations of observational studies that might lead to spurious precision of combined effect estimates. The main assumption of this method is that every observational study has a probability *c* (credibility ceiling) that the true effect size is in a different direction from the one suggested by the point estimate. This was measured by the last credibility ceiling at which the summary effect size was still statistically significant.

*Evaluating the quality of the evidence by AMSTAR-2*

To date there is no quality assessment tool specifically designed for umbrella reviews. We used the AMSTAR 2 tool[11] as a proxy to assess the methodological quality as detailed in previous umbrella reviews[12]. The AMSTAR-2 tool uses 16 measures to appraise the methodological quality of systematic reviews and ranks them into high, moderate, low or critically low quality. Seven of the sixteen measures are classed as major components, with unsatisfactory answers to any of these being regarded as a critical flaw. Assessed criteria includes a published protocol, a comprehensive literature search, a list of excluded studies, satisfactory risk of bias assessment for each included study, appropriate statistical analysis, adequate discussion regarding any bias detected and an appropriate investigation of publication bias. By assessing these criteria four grades are reached: ‘high’ to ‘critically low’ with ‘high’ having zero or one non-critical weakness in the study and ‘critically low’ having more than one critical flaw with or without non-critical weaknesses. As the AMSTAR 2 tool aims to evaluate systematic reviews of randomised and non-randomised trials, some of the criteria were not applicable to umbrella reviews.
