# Supplementary material for: Risk factors for human papillomavirus infection, cervical intraepithelial neoplasia and cervical cancer: an umbrella review and follow-up Mendelian randomisation studies
Source: BMC Med. 2023 Jul 27;21:274. doi: 10.1186/s12916-023-02965-w (PMC10375747; doi:10.1186/s12916-023-02965-w)
Supplement: Supplementary file 2 — Additional file 2: Supplementary Table 1. Statistical criteria for grading of the evidence. [file 12916_2023_2965_MOESM2_ESM.pdf]

SUPPLEMENTARY TABLES

Table S1: Statistical criteria for grading of the evidence

**Evidence grading criteria**
**For strong evidence:**
$P<10^{-6}$
>1,000 cases
P<0.05 of the largest study in a meta-analysis
$I^2<50\%$
no small study effect*
prediction interval excludes the null value
no excess significance bias**
**For highly suggestive evidence:**
$P<10^{-6}$
>1,000 cases
P<0.05 of the largest study in a meta-analysis
**For suggestive evidence:**
$P<10^{-3}$
>1,000 cases
**For weak evidence:**
P<0.05

P= p-value of random effects model

* Egger's P <0.10 signifies no small study effect

** when comparing observed and expected number of studies
