# Supplementary material for: Risk factors for human papillomavirus infection, cervical intraepithelial neoplasia and cervical cancer: an umbrella review and follow-up Mendelian randomisation studies
Source: BMC Med. 2023 Jul 27;21:274. doi: 10.1186/s12916-023-02965-w (PMC10375747; doi:10.1186/s12916-023-02965-w)
Supplement: Supplementary file 3 — Additional file 3: Supplementary Table 2. Summary quality assessment for all included systematic reviews using the ASMTAR 2 tool (A Measurement Tool to Assess Systematic Reviews 2). [file 12916_2023_2965_MOESM3_ESM.pdf]

Table S2: Summary quality assessment for all included systematic reviews using the ASMTAR 2 tool (A Measurement Tool to Assess Systematic Reviews 2).

**AMSTAR 2 Questions** / **Study Author, year**	PICO	'A priori' design and deviations justified	Study design	Literature search	Duplicate study selection review	Duplicate data extraction	Excluded studies	Description of included studies	Risk of bias assess ment	Funding	Statistical methods for meta-analysis	Impact of RoB from meta-analysis	Risk of bias of individual studies	Heterogeneity	Small study bias	Conflict of interest	**Overall rating**
Wang 2020	●	◎	●	◎	○	●	○	○	◎	○	●	○	●	●	○	●	critically low
Xie 2018	●	●	●	◎	○	●	●	◎	◎	○	○	●	●	○	●	●	low
Yang 2018	○	○	●	◎	○	○	●	●	○	○	NA	NA	○	●	○	●	critically low
Ye 2018	●	○	●	◎	●	●	●	●	○	○	●	●	○	●	●	●	critically low
Zard 2014	●	◎	●	◎	●	●	●	◎	○	○	○	○	○	○	●	●	critically low
Zeng 2012	●	○	●	◎	●	○	○	◎	◎	○	○	●	●	●	●	●	critically low
Zhang 1997	●	○	○	○	○	○	○	○	◎	○	○	○	●	○	○	○	critically low
Zhang 2012	●	◎	●	◎	○	●	○	◎	◎	○	●	●	○	●	●	●	low
Zhang 2018	●	◎	●	◎	●	●	○	◎	○	○	○	●	○	●	●	●	critically low
Allegreti 2015	●	●	●	◎	●	●	○	◎	◎	○	●	●	●	●	●	○	moderate
Appleby 2005	●	◎	○	◎	○	○	○	◎	○	○	●	●	●	●	○	○	critically low
Zhu 2016	●	●	●	◎	○	●	○	◎	◎	○	●	●	●	●	●	●	moderate
Appleby 2006	○	○	○	○	○	○	○	◎	◎	○	●	○	○	●	○	○	critically low
Hu 2017	●	●	●	◎	●	○	○	◎	○	○	○	●	●	●	●	○	critically low
Cao 2014	●	○	○	◎	○	●	○	◎	◎	○	●	●	○	●	○	○	critically low
Cao 2016	●	◎	●	◎	●	○	○	◎	○	○	●	●	●	●	●	○	critically low

Castellsague 2011	●	○	○	○	○	○	○	●	○	●	●	●	●	●	○	●	critically low
Cortessis 2017	●	◎	●	◎	●	○	○	●	◎	○	●	●	●	●	●	○	critically low
Debeaudrap 2019	●	◎	○	●	●	●	○	●	◎	○	●	●	●	●	●	●	critically low
De Lima 2018	●	○	●	◎	●	○	○	●	◎	○	●	●	●	●	○	○	critically low
Gillet 2011	●	○	●	◎	●	○	○	◎	◎	○	●	●	●	●	●	●	critically low
Gillet 2012	●	○	○	◎	○	○	○	◎	●	○	●	●	●	●	●	●	critically low
Grulich 2007	●	○	○	◎	●	○	○	◎	◎	○	●	○	○	●	●	●	critically low
Haverkos 2003	●	◎	○	◎	○	○	●	◎	◎	○	○	●	●	○	○	○	critically low
He 2017	●	◎	●	◎	○	●	○	◎	◎	○	○	●	○	●	●	●	critically low
Helm 2013	●	◎	●	◎	●	●	●	●	●	○	●	●	●	●	●	●	high
Josyula 2015	●	○	●	◎	○	○	○	◎	◎	○	●	●	○	●	●	●	critically low
Kaderli 2014	●	○	○	○	○	○	○	◎	◎	○	○	○	●	○	○	○	critically low
Kelly 2018	●	◎	○	●	○	○	○	●	◎	○	●	●	○	●	●	●	low
Lee 2016	○	◎	○	○	○	○	○	●	●	○	●	●	●	●	●	○	critically low
Li 2013	●	◎	●	◎	●	●	○	●	●	○	●	●	●	●	●	●	moderate
Liu 2014	●	◎	●	◎	○	●	●	●	●	○	●	●	●	●	●	●	moderate
Liu 2015	●	◎	●	◎	○	●	○	◎	◎	○	●	●	●	●	●	○	low
Liu 2018	●	◎	●	◎	○	○	○	◎	●	○	●	●	●	●	●	●	low
Looker 2018	○	◎	○	◎	○	●	○	◎	●	○	○	●	●	●	●	●	critically low
Manhart 2002	●	◎	●	○	○	○	○	◎	●	○	●	●	●	●	○	○	critically low
Myung 2011	●	◎	○	◎	●	○	○	○	○	○	●	○	●	●	○	●	critically low
Naldini 2019	●	◎	●	◎	●	●	○	●	◎	○	●	●	●	●	●	●	low

Peng 2017	●	◎	●	◎	●	●	○	●	●	○	●	●	●	●	●	●	critically low
Plummer 2003	○	○	○	○	○	○	○	◎	◎	○	○	○	●	●	●	○	critically low
Simon 2015	●	◎	●	◎	○	○	○	○	○	○	●	●	●	●	○	●	critically low
Smith 2003	○	○	○	○	○	○	○	●	◎	○	●	●	●	●	●	●	critically low
Tamarelle 2018	●	○	●	◎	○	○	○	○	●	○	●	●	●	●	●	●	critically low
WCRF Cup Cervical Cancer	○	◎	○	○	○	○	○	●	◎	○	●	●	●	●	●	○	critically low
Brusselaers 2019	●	●	●	●	●	●	●	◎	◎	○	●	●	●	●	○	●	moderate
Appleby 2007	●	○	●	○	○	○	○	◎	◎	○	●	○	○	●	○	●	critically low
Chen 2020	●	○	●	●	●	●	○	●	◎	○	●	○	○	●	○	●	critically low
Tomita 2020	●	●	●	●	●	●	●	●	◎	○	●	○	○	●	●	●	low
Liang 2019	●	○	●	◎	○	○	○	○	◎	○	●	○	○	●	●	●	critically low
Wang 2019	●	●	●	●	○	○	○	●	●	●	○	●	○	●	○	●	critically low

Key: ● yes, ◎ partial yes, ○ no, Critical flaw
