# Supplementary material for: Risk factors for human papillomavirus infection, cervical intraepithelial neoplasia and cervical cancer: an umbrella review and follow-up Mendelian randomisation studies
Source: BMC Med. 2023 Jul 27;21:274. doi: 10.1186/s12916-023-02965-w (PMC10375747; doi:10.1186/s12916-023-02965-w)
Supplement: Supplementary file 4 — Additional file 4: Supplementary Table 3. Description of 87 meta-analyses investigating risk factors associated with HPV, cervical pre cancer and cancer outcomes - only cohort studies included. [file 12916_2023_2965_MOESM4_ESM.pdf]

Table S3: Description of 87 meta-analyses investigating risk factors associated with HPV, cervical pre cancer and cancer outcomes - only cohort studies included.

Author, year	Exposure	Exposure contrast	Outcome	N[a]	N cohorts	N cases/cohort	Summary relative risk (95% CI)			Fixed P-value[b]	Random P-value[c]	95% Prediction interval[d]
							Fixed Effects	Random Effects	Largest Study			
Appleby 2006	Age during FTP	Per 1-year decrease	CIN3 incidence	15	5	620/1782	1.03(0.98-1.07)	1.03(0.98-1.07)	1.04(0.99-1.1)	2.38E-01	2.38E-01	0.96-1.10
WCRF CUP	Alcohol intake	Highest vs lowest	Cervical cancer incidence	4	4	1549/2686978	1.00(0.87-1.16)	1.00(0.87-1.17)	1.00(0.84-1.19)	9.56E-01	9.56E-01	0.73-1.39
Gillet 2012	Bacterial vaginosis	Yes vs no	CIN prevalence	20	12	109572/1652847	1.28(1.23-1.33)	1.62(1.30-2.04)	1.19(1.13-1.26)	6.26E-31	2.43E-05	0.78-3.39
Gillet 2011	Bacterial vaginosis	Yes vs no	HPV prevalence	12	2	199/667	2.12(1.50-3.00)	1.94(1.08-3.47)	2.42(1.64-3.57)	1.95E-05	2.67E-02	NA
WCRF CUP 2018	BMI	Highest vs lowest level	Cervical cancer mortality	4	4	420/3144461	1.64(1.25-2.15)	1.82(1.07-3.1)	1.15(0.79-1.7)	3.89E-04	2.75E-02	0.22-15.20
WCRF CUP 2018	BMI	Highest (>30) vs lowest (<25)	Cervical cancer incidence	7	7	4837/7875703	1.14(0.99-1.32)	1.13(0.9-1.380)	1.02(0.8-1.31)	7.40E-02	2.31E-01	0.71-1.79
WCRF CUP 2018	BMI	per 5kg/m2 increase	Cervical cancer incidence	9	9	5035/9680798	1.02(1.00-1.04)	1.02(0.97-1.07)	0.98(0.95-1.02)	4.22E-02	4.45E-01	0.09-1.17
WCRF CUP 2018	BMI	per 5kg/m2 increase	Cervical cancer incidence	7	7	4837/7875703	1.01(0.99-1.03)	1.01(0.96-1.06)	0.98(0.95-1.02)	3.61E-01	6.72E-01	0.87-1.17
WCRF CUP 2018	BMI	per 5kg/m2 increase	Cervical cancer mortality	3	3	307/3032760	1.02(0.90-1.14)	1.02(0.83-1.25)	1.04(0.88-1.24)	7.87E-01	8.35E-01	0.11-9.52
Wang 2019	Cervicovaginal lactobacilli	LP CSTs vs non LP CSTs	HR HPV incidence	9	2	51/104	0.68(0.29-1.59)	0.58(0.16-2.14)	0.98(0.37-2.64)	3.76E-01	4.16E-01	NA

Wang 2019	Cervicovaginal lactobacilli	LIP CST III vs non LIP CST III	HR HPV incidence	8	2	16/83	0.57(0.18-1.82)	0.57(0.18-1.82)	0.76(0.16-3.58)	3.40E-01	3.40E-01	NA
Wang 2019	Cervicovaginal lactobacilli	LCP CST I vs non LCP CST I	HR HPV incidence	8	3	59/155	0.57(0.24-1.35)	0.57(0.24-1.35)	0.5(0.18-1.39)	2.03E-01	2.03E-01	0-156.11
Zhu 2016	Chlamydia tr and HPV, coinfection	Yes vs no	Cervical cancer incidence (CIN1+)	6	3	492/2528	3.29(2.47-4.4)	3.3(2.47-4.4)	3.23(2.39-4.35)	6.42E-26	6.42E-16	NA
Naldin 2019	Chlamydia tr infection	Yes vs no	HPV incidence	40	6	2753/7426	1.51(1.07-1.24)	1.74(1.28-2.38)	1.2(1.09-1.32)	6.81E-05	4.94E-04	0.64-4.73
Naldin 2019	Chlamydia tr infection	Yes vs no	HR HPV incidence	15	2	912/2878	2.47(1.23-4.99)	2.07(1.57-2.72)	1.84(1.36-2.47)	2.00E-07	1.13E-02	NA
Zhu 2016	Chlamydia tr infection	Yes vs no	Cervical cancer incidence (CIN3+)	3	3	832/4305	2.22(1.88-2.62)	2.21(1.62-3.03)	2.21(1.84-2.65)	2.63E-21	6.43E-07	0.09-53.4
Zhu 2016	Chlamydia tr infection	Yes vs no	Cervical cancer incidence (SCC)	11	2	500/3077	2.34(1.92-2.85)	2.33(1.90-2.87)	2.4(1.95-2.94)	5.52E-17	6.59E-16	NA
Zhu 2016	Chlamydia tr infection	Yes vs no	Cervical cancer incidence (CIN1+)	11	4	875/4718	1.86(0.91-3.79)	1.86(0.91-3.79)	1.9(0.82-4.29)	8.70E-01	8.70E-01	0.39-8.85
Appleby 2007	COCP (current users)	Per year of use	Invasive cervical cancer incidence	3	3	45/311	1.03(0.97-1.09)	1.03(0.97-1.09)	1.01(0.94-1.09)	3.86E-01	3.86E-01	0.69-1.53
Appleby 2007	COCP (1-9ys since last use)	Per year of use	Invasive cervical cancer incidence	3	3	50/252	1.03(0.98-1.08)	1.03(0.98-1.09)	1.04(0.97-1.11)	2.30E-01	2.74E-01	0.65-1.64
Appleby 2007	COCP (>10ys since last use)	Per year of use	Invasive cervical cancer incidence	3	3	120/1591	1.01(0.97-1.04)	1.01(0.98-1.04)	1.00(0.96-1.04)	7.59E-01	7.59E-01	0.81-1.25
Smith 2003	COCP	<5 years users vs Never	Cervical cancer incidence (CIN3+)	5	2	94/193	1.24(0.64-2.4)	1.24(0.64-2.4)	1.2(0.6-2.4)	5.26E-01	5.26E-01	NA
Smith 2003	COCP	5-9 years users vs Never	Cervical cancer incidence (CIN2+)	5	2	101/215	0.8(0.43-1.51)	0.8(0.43-1.51)	0.8(0.4-1.5)	4.89E-01	4.89E-01	NA
Smith 2003	COCP	>10 years users vs Never	Cervical cancer incidence (CIN2+)	3	2	129/228	1.59(0.85-3.97)	1.59(0.85-2.97)	1.5(0.8-2.9)	1.44E-01	1.44E-01	NA
Smith 2003	COCP	5-9 years users vs Never	Invasive cervical cancer incidence	21	4	448/17910	2.22(1.72-2.87)	1.95(1.138-3.36)	3.1(2.1-4.5)	1.00E-09	1.51E-02	0.18-20.80

Smith 2003	COCP	<5 years users vs Never	Invasive cervical cancer incidence	20	4	470/17983	1.82(1.40-2.35)	1.81(1.40-2.35)	2.1(1.4-3.0)	6.12E-06	6.12E-06	1.03-3.20
Smith 2003	COCP	>10 years users vs Never	Invasive cervical cancer incidence	13	3	322/16209	3.27(2.37-4.51)	3.02(1.56-5.83)	4.7(2.9-7.5)	6.91E-13	1.02E-03	0-6554
Lee 2016	Environmental tobacco smoke	Yes vs no	Cervical cancer incidence (CIN2+)	17	7	432/65031	1.26(1.03-1.55)	1.41(1.01-1.96)	0.9(0.65-1.24)	2.45E-02	4.32E-02	0.58-3.44
Wang 2020	GDM	Yes vs no	Cervical cancer incidence	3	3	63694/1163875	1.02(0.82-1.29)	1.02(0.82-1.29)	0.90(0.65-126)	8.43E-01	8.43E-01	0.23-4.49
WCRF CUP	Height	Per 5 cm increase	Cervical cancer incidence	4	4	1182/990226	1.01(0.93-1.10)	1.03(0.87-1.20)	1.01(0.92-1.11)	7.73E-01	7.65E-01	0.57-1.83
Looker 2018	HIV	HIV+ vs HIV-	HR HPV incidence	11	11	2323/8668	2.22(1.99-2.47)	2.20(1.89-2.54)	2.35(2.03-2.72)	0	3.01E-26	1.63-2.97
Looker 2018	HIV	HIV+ vs HIV-	Clearance of HPV	15	15	2977/7448	0.56(0.51-0.61)	0.53(0.43-0.64)	0.67(0.56-0.81)	2.43E-36	4.32E-10	0.26-1.06
Debeaudrap 2019	HIV	HIV+ vs HIV-	Treatment failure for any grade lesions	18	15	1339/4300	4.34(3.74-5.17)	4.90(3.89-6.18)	3.2(2.4-4.3)	0	0	2.64-9.10
Grulich 2007	HIV	HIV+ vs HIV-	Cervical cancer incidence	9	9	1184/142676	5.35(4.45-6.43)	3.98(2.24-7.05)	5.2(3.81-6.93)	0	2.32E-06	0.59-26.73
Liu 2018	HIV	HIV+ vs HIV-	CIN regression (LSIL)	2	2	1968/4554	0.68(0.56-0.81)	0.68(0.56-0.81)	0.69(0.57-0.84)	3.46E-05	3.46E-05	NA
Looker 2018	HIV	HIV+ vs HIV-	HPV incidence	15	15	1722/8342	1.71(1.48-1.98)	1.73(1.39-2.16)	1.35(0.99-1.84)	2.50E-13	1.01E-06	0.91-3.31
Looker 2018	HIV	HIV+ vs HIV-	Clearance of HR HPV	11	11	3581/9903	0.70(0.65-0.75)	0.66(0.55-0.80)	0.67(0.59-0.77)	1.58E-20	8.03E-06	0.37-1.20
Liu 2018	HIV	HIV+ vs HIV-	HPV 18 incidence	2	2	54/1050	2.56(1.17-5.60)	2.56(1.17-5.60)	3.19(1.17-8.7)	1.92E-02	1.92E-02	NA
Liu 2018	HIV	HIV+ vs HIV-	Clearance of prevalent and newly detected HPV (Any HPV)	2	2	424/696	0.43(0.32-0.57)	0.34(0.16-0.75)	0.46(0.34-0.62)	5.25E-09	7.18E-03	NA
Liu 2018	HIV	HIV+ vs HIV-	Clearance of prevalent and newly detected HPV (HR HPV)	3	3	675/1053	0.64(0.52-0.79)	0.58(0.39-0.87)	0.75(0.59-0.96)	1.89E-05	7.83E-03	0.01-44.81

Liu 2018	HIV	HIV+ vs HIV-	Clearance of prevalent and newly detected HPV (HPV 16)	3	3	485/994	0.61(0.47-0.80)	0.64(0.44-0.93)	0.57(0.41-0.79)	4.23E-04	1.99E-02	0.02-20.1
Looker 2018	HIV	HIV+ vs HIV-	Clearance of HPV 16	9	9	2283/4964	0.63(0.53-0.75)	0.67(0.50-0.90)	0.56(0.39-0.81)	2.95E-07	7.28E-03	0.28-1.61
Liu 2018	HIV	HIV+ vs HIV-	HPV incidence	3	3	528/1694	2.64(2.05-3.42)	2.64(2.05-3.42)	2.98(2.07-4.29)	1.13E-13	1.13E-13	0.5-13.94
Liu 2018	HIV	HIV+ vs HIV-	HR HPV incidence	2	2	263/1210	2.35(1.64-3.37)	2.35(1.64-3.37)	2.3(1.57-3.37)	3.62E-06	3.62E-06	NA
Liu 2018	HIV	HIV+ vs HIV-	HPV 16 incidence	2	2	91/1064	3.05 (1.71-5.43)	3.047 (1.711-5.426)	3.09(1.39-6.86)	1.55E-04	1.55E-04	NA
Liu 2018	HIV	HIV+ vs HIV-	Clearance of prevalent and newly detected HPV (HR HPV)	2	2	617/1992	0.56(0.45-0.69)	0.53(0.37-0.75)	0.6(0.47-0.76)	9.75E-08	3.86E-04	NA
Liu 2018	HIV	HIV+ vs HIV-	Clearance of prevalent and newly detected HPV (HPV 18)	2	2	348/624	0.48(0.32-0.72)	0.48(0.32-0.72)	0.5(0.31-0.81)	4.31E-04	4.31E-04	NA
Liu 2018	HIV	HIV+ vs HIV-	CIN incidence (LSIL)	2	2	362/2744	3.73(2.62-5.32)	3.73(2.62-5.32)	4(2.61-6.13)	3.46E-13	3.46E-13	NA
Debeaudrap 2019	HIV	HIV+ vs HIV-	Treatment failure for high grade lesions	10	9	304/2555	2.47(1.83-3.35)	2.57(1.83-3.35)	3.5(2-6.1)	4.32E-09	4.32E-09	1.72-3.56
Liu 2018	HIV	HIV+ vs HIV-	Clearance of prevalent and newly detected HPV (HPV 16)	2	2	105/121	0.88(0.49-1.59)	0.82(0.21-3.20)	1.61(0.74-3.51)	6.82E-01	7.72E-01	NA
Looker 2018	HIV	HIV+ vs HIV-	Clearance of HPV 18	7	7	2170/4523	0.78(0.62-0.97)	0.77(0.52-1.15)	0.91(0.62-1.33)	2.89E-02	2.06E-01	0.24-2.48
Looker 2018	HIV	HIV+ with CD4>200 vs HIV-	HPV incidence	5	5	1151/2335	2.89(2.55-3.27)	3.09(2.17-4.40)	3.61(3.01-4.32)	0	3.75E-10	0.91-10.55
Looker 2018	HIV	HIV+ with CD4</=200 vs HIV-	HPV incidence	3	3	703/1212	5.33(4.48-6.34)	5.76(3.65-9.08)	5.19(4.3-6.26)	0	4.73E-14	0.04-844.24
Looker 2018	HIV	HIV+ with CD4>200 vs HIV-	HR HPV incidence	4	4	327/758	1.94(1.45-2.61)	2.03(1.3-3.17)	1.41(0.89-2.24)	8.18E-06	1.78E-03	0.36-11.36
Looker 2018	HIV	HIV+ with CD4</=200 vs HIV-	HR HPV incidence	2	2	152/334	1.32(0.82-2.08)	1.31(0.82-2.08)	1.26(0.77-2.06)	2.62E-01	2.62E-01	NA

Looker 2018	HIV	HIV+ with CD4>200 vs HIV-	Clearance of HR HPV	4	4	299/468	1.23(0.97-1.56)	1.19(0.89-1.58)	1.49(1.09-2.04)	8.30E-01	2.40E-01	0.51-2.77
Debeaudrap 2019	HIV	HIV+ vs HIV-	Treatment failure for any grade lesions	18	15	1339/4300	4.34(3.74-5.17)	4.90(3.89-6.18)	3.2(2.4-4.3)	0	0	2.64-9.10
Kelly 2018	HIV+	On ART vs no ART	CIN regression (SIL)	10	10	3074/10241	1.57(1.38-1.78)	1.62(1.32-1.99)	1.3(1-1.7)	1.38E-11	4.41E-06	0.93-2.82
Kelly 2018	HIV+	On ART vs no ART	CIN incidence (SIL)	12	11	1826/6475	0.70(0.57-0.85)	0.72(0.54-0.95)	0.62(0.42-0.91)	4.27E-04	2.15E-02	0.35-1.49
Kelly 2018	HIV+	On ART vs no ART	CIN progression (SIL)	10	10	3493/8568	0.70(0.63-0.78)	0.74(0.61-0.90)	0.66(0.54-0.81)	3.94E-10	2.63E-03	0.42-1.31
Kelly 2018	HIV+	On ART vs no ART	Invasive cervical cancer incidence	3	2	2340/411779	0.44(0.27-0.74)	0.40(0.18-0.87)	0.5(0.29-0.87)	1.64E-03	2.03E-02	NA
Liu 2018	HIV+	CD4 <200 vs >500	Clearance of HPV	2	2	812/2218	0.46(0.37-0.57)	0.46(0.37-0.57)	0.47(0.38-0.59)	1.11E-12	1.11E-12	NA
Liu 2018	HIV+	CD4 200-500 vs >500	Clearance of HPV	2	2	812/2218	0.76(0.64-0.89)	0.76(0.64-0.89)	0.76(0.64-0.9)	1.01E-03	1.01E-03	NA
Liu 2018	HIV+	On ART vs no ART	CIN regression (LSIL)	3	3	522/1604	0.65(0.52-0.82)	0.65(0.52-0.82)	0.66(0.47-0.92)	2.78E-04	2.78E-04	0.16-2.91
Kelly 2018	HIV+	On ART vs no ART	HR -HPV prevalence	20	19	3901/7996	0.76(0.70-0.83)	0.83(0.68-1.01)	0.83(0.74-0.94)	6.77E-11	6.65E-02	0.4-1.71
Kelly 2018	HIV+	On ART vs no ART	Cervical cancer prevalence (HSIL, CIN2+)	14	7	886/3724	0.72(0.54-0.96)	0.76(0.49-1.18)	0.54(0.32-0.91)	2.52E-01	2.20E-01	0.22-2.62
Liu 2018	HIV+	On ART vs no ART	CIN incidence (LSIL)	2	2	358/1254	0.67(0.45-0.99)	0.70(0.40-1.25)	0.55(0.34-0.89)	4.54E-02	2.30E-01	NA
Liu 2018	HIV+	On ART vs no ART	CIN regression (LSIL)	2	2	325/1149	2.29(1.56-3.37)	1.11(0.16-7.61)	2.61(1.75-3.89)	2.57E-05	9.13E-01	NA
Allegreti 2015	IBD on immunosuppressive medications	Yes vs healthy controls	Cervical cancer incidence	8	5	10829/244724	1.33(1.27-1.39)	1.33(1.27-1.39)	1.35(1.28-1.43)	7.78E-37	7.78E-37	1.24-1.43
Li 2013	IVF	Yes vs no	Cervical cancer incidence	4	4	33735/1453927	0.63(0.55-0.74)	1.07(0.45-2.55)	0.61(0.52-0.71)	5.01E-09	8.71E-01	0.03-34.71
Appleby 2006	Parity	Per increase of 1 FTP	CIN3 incidence	15	5	957/3209	1.10(0.99-1.20)	1.05(0.92-1.21)	1.22(1.06-1.41)	5.42E-02	4.08E-01	0.72-1.56
Liu 2014	Pregnancy	Pregnant vs non pregnant	HPV incidence	14	14	1474/9518	1.43(1.26-1.62)	1.42(1.03-1.96)	0.87(0.66-1.14)	2.86E-08	3.33E-02	0.42-4.80
Helm 2013	Retinoid use	Yes vs no	Complete or partial regression of CIN2/3 at 3-12 months	3	3	186/314	0.98(0.56-1.72)	0.98(0.56-1.72)	0.92(0.39-2.16)	9.50E-01	9.50E-01	0.03-36.66
Helm 2013	Retinoid use	Yes vs no	Complete regression of CIN2/3 at 9-27 months	2	2	160/406	0.79(0.51-1.23)	0.79(0.51-1.23)	0.71(0.41-1.24)	2.94E-01	2.94E-01	NA

Helm 2013	Retinoid use	Yes vs no	Complete regression of CIN2 at 9-27 months	2	2	116/260	0.74(0.42-1.29)	0.81(0.29-2.28)	0.5(0.25-1.02)	2.86E-01	6.93E-01	NA
Simon 2015	Rheumatoid arthritis	Yes vs general population	Cervical cancer incidence	15	15	297/297887	0.86(0.84-0.89)	0.85(0.73-0.99)	0.86(0.84-0.89)	4.19E-26	4.73E-02	0.54-1.34
Liu 2015	Sexual partners	Multiple vs few partners	CIN incidence	26	2	345/1345	1.15(0.70-1.90)	1.15(0.69-1.90)	1.18(0.6-1.75)	5.90E-01	5.90E-01	NA
Kaderli 2014	Smoking	Yes vs no	HPV incidence	10	10	9442/28187	1.33(1.21-1.46)	1.37(1.21-1.56)	1.2(1-1.3)	2.51E-09	1.04E-06	1.06-1.77
Appleby 2005	Smoking	Past vs never smoker	Cervical cancer incidence (CIN3+)	19	4	798/2873	1.54(1.13-2.09)	1.53(1.12-2.10)	1.83(1.19-2.82)	6.14E-03	8.10E-03	0.73-3.2
Kaderli 2014	Smoking	Yes vs no	HPV prevalence	5	5	4451/10853	1.19(1.08-1.33)	1.24(1.03-1.50)	1.1(1-1.4)	4.07E-04	2.63E-02	0.66-2.31
Appleby 2005	Smoking	Current vs Never	Cervical cancer incidence (CIN3+)	19	4	798/2874	1.85(1.47-2.34)	1.85(1.47-2.34)	1.68(1.17-2.41)	2.22E-07	2.22E-07	1.11-3.09
Grulich 2007	Transplant recipient	Yes vs no	Cervical cancer incidence	3	3	24/18800	2.13(1.38-3.3)	2.13(1.38-3.3)	2.5(1.33-4.27)	7.09E-04	7.09E-04	0.12-36.38
Brusselaers 2019	Vaginal dysbiosis	Yes vs no	Progression to dysplasia and CIN	9	9	27405/460746	1.58(1.42-1.75)	1.59(1.40-1.82)	1.44(1.22-1.71)	2.55E-18	5.34E-12	1.2- 2.12
Brusselaers 2019	Vaginal dysbiosis	Yes vs no	HPV incidence	4	4	1926/ 5280	1.33(1.18-1.50)	1.33(1.18-1.49)	1.24(1.04-1.47)	2.24E-06	2.24E-06	1.03-1.73
Brusselaers 2019	Vaginal dysbiosis	Yes vs no	HPV incidence	7	7	1719/4711	1.07(1.02-1.12)	1.14(1.01-1.28)	1.03(0.97-1.09)	1.08E-02	3.20E-02	0.86-1.5
Tamarelle 2018	VMB	LL-VMB vs HL-VMB	HPV incidence	7	4	1661/6995	1.65(1.38-1.96)	1.60(1.22-2.10)	2.11(1.54-2.91)	1.94E-08	7.27E-04	0.75-3.39
Tamarelle 2018	VMB	LL-VMB vs HL-VMB	HPV incidence	13	2	113/146	1.99(0.91-4.36)	1.99(0.91-4.36)	1.91(0.83-4.39)	8.51E-02	8.51E-02	NA

Abbreviations: FTP: full term pregnancy; NA: Not available; LP: lactobacillus predominant; CSTs: community state types; HPV: human papilloma virus; HR HPV: high risk HPV; LIP: Lactobacillus iners predominant; LCP: Lactobacillus crispatus predominant; chlamydia tr: chlamydia trachomatis; CIN: cervical intraepithelial neoplasia; SCC: squamous cell carcinoma; COCP: combined oral contraceptive pill; ys: years; GDM: gestational diabetes mellitus; HIV: human immunosufficiency virus; ART: antiretroviral treatment; SIL: squamous intraepithelial lesion; HSIL high grade SIL; LSIL: low grade SIL; IBD: inflammatory bowel disease; IVF: in vitro fertilization; VMB: vaginal microbiome; LL-VMB: Low lactobacillus vaginal microbiome; HL-VMB: high lactobacillus vaginal microbiome.

Key: [a] Number of studies, [b] P value of summary fixed effects estimate, [c] P value of summary random effects estimate, [d] Prediction intervals are reported only for meta-analyses including at least 3 studies
All statistical tests were two-sided
