# Supplementary material for: Risk factors for human papillomavirus infection, cervical intraepithelial neoplasia and cervical cancer: an umbrella review and follow-up Mendelian randomisation studies
Source: BMC Med. 2023 Jul 27;21:274. doi: 10.1186/s12916-023-02965-w (PMC10375747; doi:10.1186/s12916-023-02965-w)
Supplement: Supplementary file 5 — Additional file 5: Supplementary Table 4. Evaluation of heterogeneity, small study effects and excess significance bias in the 87 meta-analyses investigating the risk factors associated with HPV, cervical pre cancer and cancer outcomes - only cohort studies included. [file 12916_2023_2965_MOESM5_ESM.pdf]

Table S4: Evaluation of heterogeneity, small study effects and excess significance bias in the 87 meta-analyses investigating the risk factors associated with HPV, cervical pre cancer and cancer outcomes - only cohort studies included.

Author, year	Exposure	Exposure contrast	Outcome	Egger's P[a]	I^2 (95% CI) P[b]	Observed	Expected [c], P-value[d]					
							Fixed effects		Random effects		Largest study	
Appleby_2006	Age during FTP	Per 1-year decrease	CIN3	0.54	0(0-64)0.46	0	0.26	NP	0.26	NP	0.27	NP
WCRF CUP	Alcohol intake	Highest vs lowest	Cervical cancer incidence	0.14	0(0-68)0.99	0	0.20	NP	0.20	NP	0.20	NP
Gillet_2012	Bacterial vaginosis	Yes vs no	CIN prevalence	0.20	92(88-94)0	8	5.19	0.10	8.20	NP	4.33	0.03
Gillet 2011	Bacterial vaginosis	Yes vs no	HPV prevalence	NA	51(1.08-3.47)0.15	1	1.69	NP	1.53	NP	1.84	NP
WCRF CUP	BMI	Highest vs lowest level	Cervical cancer mortality	0.63	65(0-86)0.04	2	2.93	NP	3.35	NP	0.50	0.02
WCRF CUP	BMI	Highest (>30) vs lowest (<25)	Cervical cancer incidence	0.94	33(0-71)0.18	1	2.52	NP	2.34	NP	0.41	0.35
WCRF CUP	BMI	per 5kg/m2 increase	Cervical cancer incidence	0.79	72(32-84)<0.01	2	0.53	0.04	0.51	0.03	0.52	0.04
WCRF CUP	BMI	per 5kg/m2 increase	Cervical cancer incidence	0.96	68(3-84)<0.01	1	0.37	0.29	0.37	0.29	0.42	0.36
WCRF CUP	BMI	per 5kg/m2 increase	Cervical cancer mortality	0.86	65(0-88)<0.01	0	0.15	NP	0.16	NP	0.17	NP
Wang 2019	Cervicovaginal lactobacilli	LP CSTs vs non LP CST IV	HR HPV incidence	NA	49(0.16-2.14)0.16	0	0.21	NP	0.33	NP	0.10	NP
Wang 2019	Cervicovaginal lactobacilli	LIP CST III vs non LIP CST III	HR HPV incidence	NA	0(0.18-1.82)0.58	0	0.24	NP	0.24	NP	0.13	NP
Wang 2019	Cervicovaginal lactobacilli	LCP CST I vs non LCP CST I	HR HPV incidence	0.19	0(0-73)0.89	0	0.48	NP	0.48	NP	0.65	NP
Zhu_2016	Chlamydia tr and HPV, coinfection	Yes vs no	Cervical cancer incidence (CIN1+)	NA	0(2.47-4.4)0.63	2	1.98	0.89	1.98	0.89	1.98	0.89
Naldin_2019	Chlamydia tr infection	Yes vs no	HPV incidence	0.04	90(82-94)0	5	1.42	<0.01	5.73	0.16	2.07	<0.01
Naldin_2019	Chlamydia tr infection	Yes vs no	HR HPV incidence	NA	73(1.23-4.99)0.06	2	2	NP	2.0	NP	1.98	0.88
Zhu_2016	Chlamydia tr infection	Yes vs no	Cervical cancer incidence (CIN3+)	0.96	46(1.62-3.03)0.16	2	2.86	NP	2.86	NP	2.86	NP

Zhu_2016	Chlamydia tr infection	Yes vs no	Cervical cancer incidence (SCC)	NA	0.9(1.9-2.87)0.32	1	1.83	NP	1.83	NP	1.83	NP
Zhu_2016	Chlamydia tr infection	Yes vs no	Cervical cancer incidence (CIN1+)	0.59	0(0.91-3.79)0.99	0	3.19	NP	3.19	NP	3.26	NP
Appleby_2007	COCP (current users)	Per year of use	Invasive cervical cancer incidence	0.86	0(0.97-1.1)0.55	0	0.15	NP	0.15	NP	0.153	NP
Appleby 2007	COCP (1-9ys since last use)	Per year of use	Invasive cervical cancer incidence	0.56	19(0-78)0.29	0	0.15	NP	0.15	NP	0.15	NP
Appleby_2007	COCP (>10ys since last use)	Per year of use	Invasive cervical cancer incidence	NA	0(0-73)0.88	0	0.15	NP	0.15	NP	0.15	NP
Smith_2003	COCP	<5 years of use vs Never	Cervical cancer incidence (CIN3+)	NA	0(0.64-2.4)0.77	0	0.18	NP	0.18	NP	0.16	NP
Smith_2003	COCP	5-9 years of usevs Never	Cervical cancer incidence (CIN2+)	NA	0(0.43-1.51)1	0	0.19	NP	0.19	NP	0.19	NP
Smith_2003	COCP	>10 years of use vs Never	Cervical cancer incidence (CIN2+)	NA	0(0.85-2.97)0.47	0	0.52	NP	0.52	NP	0.43	NP
Smith_2003	COCP	5-9 years of usevs Never	Invasive cervical cancer incidence	0.18	75(0-89)<0.01	3	3.76	NP	3.49	NP	3.98	NP
Smith_2003	COCP	<5 years of use vs Never	Invasive cervical cancer incidence	0.23	0(1.4-2.35)0.57	2	3.04	0.22	3.04	0.22	3.47	0.03
Smith_2003	COCP	>10 years of use vs Never	Invasive cervical cancer incidence	0.36	75(1.56-5.83)0.02	2	2.99	NP	2.99	NP	3.0	NP
Lee_2016	Environmental tobacco smoke	Yes vs no	Cervical cancer incidence (CIN2+)	0.19	52(0-78)0.05	2	1.99	0.99	2.70	NP	1.44	0.60
Wang 2020	GDM	Yes vs no	Cervical cancer incidence	0.04	0(0-73)0.55	0	1.14	NP	1.14	NP	3	NP
WCRF CUP	Height	Per 5 cm increase	Cervical cancer incidence	0.76	44(0-80)0.15	0	0.21	NP	0.22	NP	0.20	NP
Looker_2018	HIV	HIV+ vs HIV-	HR HPV incidence	0.77	22(0-61)0.2316	10	9.43	0.62	9.38	0.60	9.68	0.77
Looker_2018	HIV	HIV+ vs HIV-	Clearance of HPV	0.37	73(52-83)0	8	11	0.13	1.15	<0.01	7.35	0.74
Debeaudrap_2019	HIV	HIV+ vs HIV-	Treatment failure for any grade lesions	<0.01	38(0-65)0.07	14	14.5	0.42	1.47	0.22	1.36	0.74
Grulich_2007	HIV	HIV+ vs HIV-	Cervical cancer incidence	0.30	86(75-91)0	5	6.19	NP	5.37	NP	6.12	NP
Liu_2018	HIV	HIV+ vs HIV-	CIN regression (LSIL)	NA	0(0.56-0.81)0.42	1	1.92	NP	1.92	NP	1.89	NP
Looker_2018	HIV	HIV+ vs HIV-	HPV incidence	0.57	47(0-70)0.02	6	8.24	NP	8.45	NP	3.64	NP
Looker_2018	HIV	HIV+ vs HIV-	Clearance of HR HPV	0.40	77(54-86)0	9	5.26	0.02	6.13	<0.01	5.96	<0.01
LIU_2018	HIV	HIV+ vs HIV-	HPV 18 incidence	NA	0(1.17-5.6)0.49	1	1.36	NP	1.36	NP	1.68	NP
Liu_2018	HIV	HIV+ vs HIV-	Clearance of prevalent and newly detected HPV (Any HPV)	NA	63(0.16-0.75)0.1	2	1.89	0.73	1.98	0.88	1.82	0.66
Liu_2018	HIV	HIV+ vs HIV-	Clearance of prevalent and newly detected	0.50	61(0-87)0.08	2	1.81	0.82	2.31	0.67	0.94	0.18

			HPV (HR HPV)									
Liu_2018	HIV	HIV+ vs HIV-	Clearance of prevalent and newly detected HPV (HPV 16)	0.37	30(0-80)0.24	1	1.90	NP	1.68	NP	2.19	NP
Looker_2018	HIV	HIV+ vs HIV-	Clearance of HPV 16	0.25	60(0-79)<0.01	4	4.55	NP	3.97	0.98	5.49	NP
Liu_2018	HIV	HIV+ vs HIV-	HPV incidence	0.97	0(0-73)0.63	2	2.91	NP	2.91	NP	2.96	NP
Liu_2018	HIV	HIV+ vs HIV-	HR HPV incidence	NA	0(1.64-3.37)0.74	1	1.82	NP	1.82	NP	1.80	NP
Liu_2018	HIV	HIV+ vs HIV-	HPV 16 incidence	NA	0(1.71-5.43)0.96	2	1.88	0.73	1.88	0.73	1.89	0.74
Liu_2018	HIV	HIV+ vs HIV-	Clearance of prevalent and newly detected HPV (HR HPV)	NA	47(0.37-0.75)0.17	2	1.49	0.41	1.56	0.45	1.39	0.35
Liu_2018	HIV	HIV+ vs HIV-	Clearance of prevalent and newly detected HPV (HPV 18)	NA	0(0.32-0.72)0.71	2	1.81	0.65	1.81	0.65	1.75	0.59
Liu_2018	HIV	HIV+ vs HIV-	CIN incidence (LSIL)	NA	0(2.62-5.32)0.57	2	2	NP	2.00	NP	2.00	NP
Debeaudrap_2019	HIV	HIV+ vs HIV-	Treatment failure for high grade lesions	0.26	0(0-54)0.80	3	5.94	NP	5.94	NP	7.58	NP
Liu_2018	HIV	HIV+ vs HIV-	Clearance of prevalent and newly detected HPV (HPV 16)	NA	81(0.21-3.2)0.02	1	1.06	NP	1.06	NP	1.14	NP
Looker_2018	HIV	HIV+ vs HIV-	Clearance of HPV 18	1.0	61(0-81)0.02	3	1.86	0.33	1.93	0.36	0.56	<0.01
Looker 2018	HIV	HIV+ with CD4>200 vs HIV-	HPV incidence	0.71	82(2.17-4.4)0	5	4.97	0.87	4.97	0.9	5.0	0.96
Looker 2018	HIV	HIV+ with CD4</=200 vs HIV-	HPV incidence	0.75	62(3.65-9.08)0.07	3	3.00	1	3.00	1.0	3.0	1.0
Looker 2018	HIV	HIV+ with CD4>200 vs HIV-	HR HPV incidence	0.40	55(1.30-3.18)0.09	2	2.61	NP	2.74	NP	1.12	0.33
Looker 2018	HIV	HIV+ with CD4</=200 vs HIV-	HR HPV incidence	NA	0(0.82-2.08)0.67	0	0.36	0.5	0.36	0.51	0.29	0.56
Looker 2018	HIV	HIV+ with CD4>200 vs HIV-	Clearance of HR HPV	0.14	19(0.89-1.58)0.3	1	0.47	0.42	0.38	0.29	1.19	NP
Debeaudrap_2019	HIV	HIV+ vs HIV-	Treatment failure for any grade lesions	<0.01	38(0-65)0.07	14	14.5	0.42	14.7	0.22	1.36	0.74
Kelly_2018	HIV+	On ART vs no ART	CIN regression (SIL)	0.38	50(0-74)0.04	6	7.13	NP	7.3	NP	5.33	0.67
Kelly_2018	HIV+	On ART vs no ART	CIN incidence (SIL)	0.48	40(0-69)0.08	3	5.06	NP	4.53	NP	6.65	NP
Kelly_2018	HIV+	On ART vs no ART	CIN progression (SIL)	0.13	60(0-78)<0.01	5	5.97	NP	5.07	NP	6.87	NP

Kelly_2018	HIV+	On ART vs no ART	Invasive cervical cancer incidence	NA	32(0.18-0.87)0.23	2	1.96	0.85	1.99	0.92	1.89	0.73
Liu_2018	HIV+	CD4 <200 VS >500	Clearance of HPV	NA	0(0.37-0.57)0.34	2	1.99	0.92	1.99	0.92	1.99	0.91
Liu_2018	HIV+	CD4 200-500 VS >500	Clearance of HPV	NA	0(0.64-0.89)0.79	1	1.20	NP	1.20	NP	1.18	NP
Liu_2018	HIV+	On ART vs no ART	CIN regression (LSIL)	0.65	0(0-73)0.94	2	1.83	0.84	1.83	0.84	1.77	0.79
Kelly_2018	HIV+	On ART vs no ART	HR-HPV prevalence	0.35	72(53-81)0	4	5.35	0.49	3.15	0.60	3.18	0.61
Kelly_2018	HIV+	On ART vs no ART	Cervical cancer prevalence (HSIL CIN2+)	0.53	53(0-78)0.05	2	2.13	NP	1.77	0.84	3.97	NP
Liu_2018	HIV+	On ART vs no ART	CIN incidence (LSIL)	NA	49(0.4-1.25)0.17	1	1.25	NP	1.04	NP	1.78	NP
Liu_2018	HIV+	On ART vs no ART	CIN regression (LSIL)	NA	84(0.16-7.61)0.01	1	1.52	NP	0.20	0.06	1.65	NP
Allegreti_2015	IBD on immunosuppressive medication	Yes vs healthy controls	HSIL+	0.29	0(0-64)0.42	3	3.41	NP	3.41	NP	3.45	NP
Li_2013	IVF	yes vs no	Cervical cancer incidence	0.29	69(0-87)0.022	2	1.40	0.53	1.16	0.35	1.44	0.56
Appleby_2006	Parity	Per increase of 1FTP	CIN3 incidence	0.11	43(0-78)0.14	1	0.43	0.36	0.32	0.21	1.11	NP
Liu_2014	Pregnancy	Pregnant vs non pregnant	HPV incidence	0.93	82(71-88)0	6	4.65	0.44	4.53	0.40	1.50	<0.01
Helm_2013	Retinoid use	Yes vs no	Complete or partial regression of CIN2/3 at 3-12 months	0.16	0(0-73)0.41	0	0.15	NP	0.15	NP	0.16	NP
Helm_2013	Retinoid use	Yes vs no	Complete regression of CIN2/3 at 9-27 months	NA	0(0.51-1.23)0.532	0	0.29	NP	0.29	NP	0.51	NP
Helm_2013	Retinoid use	Yes vs no	Complete regression of CIN2 at 9-27 months	NA	69(0.19-2.28)0.07	0	0.30	NP	0.19	NP	1.11	NP
Simon_2015	Rheumatoid arthritis	Yes vs general population	Cervical cancer incidence	0.97	63(26-78)<0.01	3	1	0.04	1.01	0.04	1.0	0.04
Liu_2015	Sexual partners	Multiple vs few partners	CIN incidence	NA	0(0.7-1.9)0.77	0	1.17	NP	1.17	NP	1.22	NP
Kaderli_2014	Smoking	Yes vs no	HPV incidence	0.48	22(0-62)0.24	4	5.91	NP	6.15	NP	4.49	NP
Appleby 2005	Smoking	Past vs never smoker	Cervical cancer incidence (CIN3+)	0.13	3(0-69)0.38	1	2.92	NP	2.89	NP	3.61	NP
Kaderli_2014	Smoking	Yes vs no	HPV prevalence	0.46	66(0-85)0.02	3	2.49	0.65	3.03	NP	2.52	0.67
Appleby 2005	Smoking	Current vs Never	Cervical cancer incidence (CIN3+)	0.84	0(0-68)0.87	2	3.64	NP	3.64	NP	3.64	NP
Grulich 2007	Transplant recipient	Yes vs no	Cervical cancer incidence	0.39	0(1.38-3.3)<0.01	1	0.66	0.63	0.66	0.63	0.89	0.89

Brusselaers 2019	Vaginal dysbiosis	Yes vs no	Progression to dysplasia and CIN	0.63	26(0-65)0.21	5	7.34	NP	7.40	NP	6.75	NP
Brusselaers_2019	Vaginal dysbiosis	Yes vs no	HPV incidence	0.31	0(0-68)0.63	3	2.76	0.79	2.76	0.79	1.97	0.30
Brusselaers_2019	Vaginal dysbiosis	Yes vs no	HPV incidence	0.23	44(0-75)0.1	3	0.51	<0.01	0.95	<0.01	0.38	<0.01
Tamarelle_2018	VMB	LL-VMB vs HL-VMB	HPV incidence	0.39	51(0-79)0.07	3	4.46	NP	4.28	NP	5.38	NP
Tamarelle_2018	VMB	LL-VMB vs HL-VMB	HPV incidence	NA	0(0.91-4.36)0.77	0	0.49	NP	0.49	NP	0.45	NP

Abbreviations: NA: Not available, due to <3 included studies; NP: Not Pertinent, because the estimated is larger than the observed, and there is no evidence of excess statistical significance based on the assumption made for the plausible effect size; LP: lactobacillus predominant; CSTs: community state types; HPV: human papilloma virus; HR HPV: high risk HPV; LIP: Lactobacillus iners predominant; LCP: Lactobacillus crispatus predominant; CIN: cervical intraepithelial neoplasia; SCC: squamous cell carcinoma; COCP: combined oral contraceptive pill; GDM: gestational diabetes mellitus; HIV: human immunosufficiency virus; ART: antiretroviral treatment; SIL: squamous intraepithelial lesion; HSIL high grade SIL; LSIL: low grade SIL; IBD: inflammatory bowel disease; IVF: in vitro fertilization; FTP: full term pregnancy; VMB: vaginal microbiome; LL-VMB: Low lactobacillus vaginal microbiome; HL-VMB: high lactobacillus vaginal microbiome.

Key: [a] P-value from the Egger's regression asymmetry test ($P<0.10$) **,** [b] I^2 metric of inconsistency (95% confidence interval) and the P-value of the Q test**,** [c] Expected number of statistically significant studies using the point estimate of each meta-analysis (from fixed effect, random effect of largest study accordingly) as the plausible effect size**,** [d] P value of the excess statistical significance test
All statistical tests were two-sided
