# Supplementary material for: Risk factors for human papillomavirus infection, cervical intraepithelial neoplasia and cervical cancer: an umbrella review and follow-up Mendelian randomisation studies
Source: BMC Med. 2023 Jul 27;21:274. doi: 10.1186/s12916-023-02965-w (PMC10375747; doi:10.1186/s12916-023-02965-w)
Supplement: Supplementary file 6 — Additional file 6: Supplementary Table 5. Details of evidence grading for meta-analysis of risk factors for HPV, cervical precancer and cancer outcomes – _only cohort studies included*. [file 12916_2023_2965_MOESM6_ESM.pdf]

Table S5: Details of evidence grading for meta-analysis of risk factors for HPV, cervical precancer and cancer outcomes – only cohort studies included*

Exposure	Exposure contrast	Outcome	N[*]	Sample size Cases/cohort	Largest study[#]	Random effects summary RR (95% CI)[¥]	Random P-value[‖]	95% Prediction interval	Egger's P[∞]	I^2 (%)	Excess significance[§]	
											O/E [α]	P-value[j]
**Strong evidence**												
HIV	HIV+ vs HIV-	HR HPV incidence	11	2323/8668	2.35(2.03-2.72)	2.2(1.89-2.54)	3.01E-26	1.63-2.97	0.77	22	10/9.7	0.77
IBD on immunosuppressive med	Yes vs healthy controls	Cervical high-grade dysplasia/cancer	5	10829/244724	1.35(1.28-1.43)	1.33(1.27-1.39)	7.78E-37	1.24-1.43	0.29	0	3/3.5	NP
Vaginal dysbiosis	Yes vs no	Progression to cervical dysplasia, CIN	9	27405/460746	1.44(1.22-1.71)	1.59(1.40-1.81)	5.34E-12	1.20- 2.12	0.63	26	5/6.8	NP
**Highly suggestive**												
HIV	Positive vs negative	HPV clearance	15	2977/7448	0.67(0.56-0.81)	0.53(0.43-0.64)	4.32E-10	0.26-1.06	0.37	73	8/7.4	0.74
HIV	HIV+, CD4>200 vs HIV-	HPV incidence	5	1151/2335	3.61(3.01-4.32)	3.09(2.17-4.40)	3.75E-10	0.91-10.55	0.709	82	5/5	0.96
HIV	HIV+ vs HIV-	Cervical cancer incidence	6	1160/122692	5.2(3.81-6.93)	5.82(2.98-11.34)	2.34E-07	0.67-50.17	0.78	86	4/4.1	NP
**Suggestive evidence**												
Chlamydia tr	Yes vs no	HPV incidence	6	2753/7426	1.2(1.09-1.32)	1.74(1.28-2.38)	4.94E-04	0.64-4.73	0.04	90	5/2.1	0.01
HIV	HIV+ vs HIV-	CIN regression (LSIL)	2	1968/4554	0.69(0.57-0.84)	0.68(0.56-0.81)	3.46E-05	NA	NA	0	1/1.9	NP
HIV	HIV+ vs HIV-	HPV incidence	15	1722/8342	1.35(0.99-1.84)	1.73(1.39-2.16)	1.01E-06	0.91-3.31	0.57	47	6/3.6	NP
HIV	HIV+ vs HIV-	Clearance of HR HPV	11	3581/9903	0.67(0.59-0.77)	0.66(0.55-0.79)	8.03E-06	0.37-1.20	0.40	77	9/6	0.07
HIV+	On ART vs not on ART	CIN regression (SIL)	10	3074/7953	1.30(1.00-1.70)	1.62(1.32-1.99)	4.41E-06	0.93-2.82	0.38	50	6/5.3	0.67
Smoking	Yes vs no	HPV incidence	10	9442/28187	1.20(1.00-1.30)	1.37(1.21-1.56)	1.04E-06	1.06-1.77	0.48	22	4/4.5	NP
Vaginal dysbiosis	Yes vs no	HPV incidence	4	1926/ 5280	1.24(1.04-1.47)	1.33(1.18-1.49)	2.24E-06	1.03-1.73	0.31	0	3/2	0.30

VMB	LL-VMB vs HL-VMB	HPV incidence	4	1661/6995	2.11(1.54-2.91)	1.60(1.22-2.10)	7.27E-04	0.75-3.39	0.39	51	3/5.4	NP
Bacterial vaginosis	Yes vs no	CIN prevalence	20	109573/1652848	1.19(1.13-1.26)	1.63(1.30-2.40)	2.43E-05	0.78-3.39	0.20	92	8/4.3	0.03
**Weak evidence**												
Bacterial vaginosis	Yes vs no	HPV prevalence	2	199/667	2.42(1.64-3.57)	1.94(1.08-3.47)	2.67E-02	NA	NA	51	1/1.8	NP
BMI	Highest vs lowest level	Cervical cancer mortality	4	420/3144461	1.15(0.79-1.70)	1.82(1.07-3.10)	2.75E-02	0.22-15.20	0.63	65	2/0.5	0.02
Chl tr-coinfection with HPV	Yes vs no	Cervical cancer incidence (CIN1+)	3	492/2528	3.23(2.39-4.35)	3.3(2.47-4.40)	6.42E-16	NA	NA	0	2/1.9	0.90
Chlamydia tr infection	Yes vs no	Cervical cancer incidence (CIN3+)	3	832/4305	2.21(1.84-2.65)	2.21(1.62-3.03)	6.43E-07	0.09-53.40	0.96	46	2/2.9	NP
Chlamydia tr infection	Yes vs no	Cervical cancer incidence (SCC)	2	500/3077	2.40(1.95-2.94)	2.33(1.90-2.87)	6.59E-16	NA	NA	0.9	1/1.8	NP
Chlamydia tr infection	Yes vs no	HPV incidence (HR)	2	912/2878	1.84(1.36-2.47)	2.07(1.57-2.72)	1.13E-02	NA	NA	73	2/1.9	0.88
COCP	<5 years user vs Never	Invasive cervical cancer incidence	4	470/17983	2.10(1.40-3.00)	1.82(1.40-2.35)	6.12E-06	1.03-3.20	0.23	0	2/3.5	0.03
COCP	5-9 years user vs Never	Invasive cervical cancer incidence	4	448/17910	3.10(2.10-4.50)	1.95(1.14-3.36)	1.51E-02	0.18-20.80	0.18	75	3/4	NP
COCP	>10 years user vs Never	Invasive cervical cancer incidence	3	322/16209	4.70(2.90-7.50)	3.02(1.56-5.83)	1.02E-03	0-6554	0.36	75	2/3	NP
Environmental tobacco smoke	Yes vs no	Cervical cancer incidence (CIN2+)	7	432/65031	0.90(0.65-1.24)	1.41(1.01-1.96)	4.32E-02	0.58-3.44	0.19	52	2/1.4	0.60
HIV	HIV+, CD4</=200 vs HIV-	HPV incidence	3	703/1212	5.19(4.30-6.26)	5.76(3.65-9.08)	4.73E-14	0.04-844.24	0.75	62	3/3	1.00
HIV	HIV+, CD4>200 vs HIV-	HR HPV incidence	4	327/758	1.41(0.89-2.24)	2.03(1.30-3.17)	1.78E-03	0.36-11.36	0.40	55	2/1.1	0.33
HIV	HIV+ vs HIV-	HPV incidence	3	528/1694	2.98(2.07-4.29)	2.64(2.05-3.42)	1.13E-13	0.50-13.94	0.96	0	2/3	NP
HIV	HIV+ vs HIV-	HR HPV incidence	2	263/1210	2.30(1.57-3.37)	2.35(1.64-3.37)	3.62E-06	NA	NA	0	1/1.8	NP
HIV	HIV+ vs HIV-	HPV 16 incidence	2	91/1211	3.09(1.39-6.86)	3.05(1.71-5.43)	1.55E-04	NA	NA	0	2/1.9	0.74
HIV	HIV+ vs HIV-	HPV 18 incidence	2	54/1211	3.19(1.17-8.70)	2.56(1.17-5.60)	1.92E-02	NA	NA	0	1/1.7	NP
HIV	HIV+ vs HIV-	Clearance** of any HPV type	2	424/696	0.46(0.34-0.62)	0.34(0.16-0.75)	7.18E-03	NA	NA	63	2/1.8	0.66
HIV	HIV+ vs HIV-	Clearance** of HR HPV	2	617/1992	0.60(0.47-0.76)	0.53(0.37-0.75)	3.86E-04	NA	NA	47	2/1.4	0.35
HIV	HIV+ vs HIV-	Clearance** of HR HPV	3	675/1053	0.75(0.59-0.96)	0.58(0.39-0.87)	7.83E-03	0.01-44.81	0.50	61	2/9.4	0.18
HIV	HIV+ vs HIV-	Clearance** of HPV 16	3	485/994	0.57(0.41-0.79)	0.64(0.44-0.93)	1.99E-02	0.02-20.10	0.37	30	1/2.2	NP
HIV	HIV+ vs HIV-	Clearance** of HPV 18	2	348/624	0.50(0.31-0.81)	0.48(0.32-0.72)	4.31E-04	NA	NA	0	2/1.8	0.59
HIV	HIV+ vs HIV-	CIN incidence (LSIL)	2	362/2744	4.00(2.61-6.13)	3.73(2.62-5.32)	3.46E-13	NA	NA	0	2/2	NP

HIV	HIV+ vs HIV-	Clearance of HPV 16	9	2283/4964	0.56(0.39-0.81)	0.67(0.50-0.90)	7.28E-03	0.28-1.61	0.25	60	4/5.5	NP
HIV+	on ART vs no ART	CIN incidence (SIL)	11	1826/6475	0.62(0.42-0.91)	0.72(0.54-0.95)	2.15E-02	0.35-1.49	0.48	40	3/6.7	NP
HIV+	on ART vs no ART	CIN progression (SIL)	10	3493/8568	0.66(0.54-0.81)	0.74(0.61-0.90)	2.63E-03	0.42-1.31	0.13	60	5/6.9	NP
HIV+	on ART vs no ART	Invasive cervical cancer incidence	2	2340/411779	0.50(0.29-0.87)	0.40(0.18-0.87)	2.03E-02	NA	NA	32	2/1.9	0.73
HIV+	CD4 <200 VS >500	Clearance of HPV	2	812/2218	0.47(0.38-0.59)	0.46(0.37-0.57)	1.11E-12	NA	NA	0	2/1.9	0.91
HIV+	CD4 200-500 VS >500	Clearance of HPV	2	812/2218	0.76(0.64-0.9)	0.76(0.64-0.89)	1.01E-03	NA	NA	0	1/1.2	NP
HIV+	on ART vs no ART	CIN incidence (LSIL)	3	522/1604	0.66(0.47-0.92)	0.65(0.52-0.82)	2.78E-04	0.16-2.91	0.65	0	2/1.8	0.79
Pregnancy	Pregnant vs non pregnant	HPV incidence	14	1474/9518	0.87(0.66-1.14)	1.42(1.03-1.96)	3.33E-02	0.42-4.80	0.93	82	6/1.5	<0.01
Rheumatoid arthritis	Yes vs general population	Cervical cancer incidence	15	297/297887	0.86(0.84-0.89)	0.85(0.73-0.99)	4.73E-02	0.54-1.34	0.97	63	2/1	0.04
Smoking	Current vs Never	Cervical cancer incidence (CIN3+)	4	798/2874	1.68(1.17-2.41)	1.85(1.47-2.34)	2.22E-07	1.11-3.09	0.84	0	2/3.6	NP
Smoking	Past vs never smoker	Cervical cancer incidence (CIN3+)	4	798/2873	1.83(1.19-2.82)	1.53(1.12-2.10)	8.10E-03	0.73-3.20	0.13	3	1/3.6	NP
Smoking	Yes vs no	HPV prevalence	5	4451/10853	1.10(1.00-1.40)	1.24(1.03-1.50)	2.63E-02	0.66-2.31	0.46	66	3/2.5	0.67
Transplant recipient	Yes vs no	Cervical cancer incidence	3	24/18800	2.50(1.33-4.27)	2.13(1.38-3.30)	7.09E-04	0.12-36.38	0.39	0	1/0.9	0.89
Vaginal dysbiosis	Yes vs no	HPV persistence	7	1719/4711	1.03(0.97-1.09)	1.14(1.01-1.28)	3.20E-02	0.86-1.50	0.23	44	3/0.4	<0.01

Abbreviations: HPV: human papilloma virus; HR HPV: high risk HPV; IBD: inflammatory bowel disease; HIV: human immunosufficiency virus; NP: Not Pertinent, because the estimated is larger than the observed, and there is no evidence of excess statistical significance based on the assumption made for the plausible effect size; CIN: cervical intraepithelial neoplasia; SIL: squamous intraepithelial lesion; HSIL high grade SIL; LSIL: low grade SIL; ART: antiretroviral treatment; VMB: vaginal microbiome; LL-VMB: Low lactobacillus vaginal microbiome; HL-VMB: high lactobacillus vaginal microbiome; Chlamydia tr: chlamydia trachomatis; SCC: squamous cell carcinoma; COCP: combined oral contraceptive pill.

Key: *Only meta-analyses meeting at least weak grade of evidence listed, [*] Number of studies, [#] Relative risk and 95% confidence interval of largest study (smallest standard error) in each meta-analysis, [¥] Random effects refer to summary risk ratio (95% confidence interval) using the random-effects model, [‖] P value of summary random effects estimate, [∞] P-value from the Egger's regression asymmetry test, [§] Expected number of statistically significant studies using the point estimate of the largest study (smallest standard error) as the plausible effect size, [α] Observed/Expected number of statistically significant studies, [j] P value of the excess statistical significance test, ** Clearance of prevalent and newly detected
All statistical tests were two-sided
