# Supplementary material for: Risk factors for human papillomavirus infection, cervical intraepithelial neoplasia and cervical cancer: an umbrella review and follow-up Mendelian randomisation studies
Source: BMC Med. 2023 Jul 27;21:274. doi: 10.1186/s12916-023-02965-w (PMC10375747; doi:10.1186/s12916-023-02965-w)
Supplement: Supplementary file 7 — Additional file 7: Supplementary Table 6. Sensitivity analysis using credibility ceilings when the association is non-significant of the 87 studies investigating the risk factors associated with HPV, cervical pre cancer and cancer outcomes - only cohort studies included. [file 12916_2023_2965_MOESM7_ESM.pdf]

Table S6: Sensitivity analysis using credibility ceilings when the association is non-significant of the 87 studies investigating the risk factors associated with HPV, cervical pre cancer and cancer outcomes - only cohort studies included.

Author, year	Exposure	Exposure contrast	Outcome	N[a]	Credibility ceiling when association non-significant
Appleby 2006	Age at FTP	Per 1-year decrease	CIN incidence	5	<1%
WCRF CUP 2018	Alcohol intake	Highest vs lowest	Cervical cancer incidence	4	<1%
Gillet 2012	Bacterial Vaginosis	Yes vs no	CIN prevalence	12	18%
Gillet 2011	Bacterial Vaginosis	Yes vs no	HPV prevalence	2	<1%
WCRF CUP 2018	BMI	Highest vs lowest level	Cervical cancer mortality	4	2%
WCRF CUP 2018	BMI	>30 vs <25	Cervical cancer incidence	7	<1%
WCRF CUP 2018	BMI	per 5kg/m2 increase of BMI	Cervical cancer incidence	9	<1%
WCRF CUP 2018	BMI	per 5kg/m2 increase of BMI	Cervical cancer incidence	7	<1%
WCRF CUP 2018	BMI	per 5kg/m2 increase of BMI	Cervical cancer mortality	3	<1%
Wang 2019	Cervicovaginal lactobacilli	LP CSTs vs non LP CST IV	HPV incidence	2	<1%
Wang_2019	Cervicovaginal lactobacilli	LIP CST III vs non LIP CST III	HPV incidence	2	<1%
Wang 2019	Cervicovaginal lactobacilli	LCP CST I vs non LCP CST I	HPV incidence	3	<1%
Zhu 2016	Chlamydia tr and HPV, coinfection	Yes vs no	Cervical cancer incidence	3	12%
Naldin 2019	Chlamydia tr infection	Yes vs no	HPV incidence	6	3%
Naldin 2019	Chlamydia tr infection	Yes vs no	HPV incidence	2	6%
Zhu 2016	Chlamydia tr infection	Yes vs no	Cervical cancer incidence	3	2%
Zhu 2016	Chlamydia tr infection	Yes vs no	Cervical cancer incidence	2	4%
Zhu_2016	Chlamydia tr infection	Yes vs no	Cervical cancer incidence	4	<1%
Smith 2003	COCP	<5 years users vs Never	Invasive cervical cancer incidence	4	10%

Smith 2003	COCP	5-9 years users vs Never	Invasive cervical cancer incidence	4	2%
Smith 2003	COCP	>10 years users vs Never	Invasive cervical cancer incidence	3	7%
Smith 2003	COCP	<5 years users vs Never	Cervical cancer incidence	2	<1%
Smith 2003	COCP	5-9 years users vs Never	Cervical cancer incidence	2	<1%
Smith_2003	COCP	>10 years users vs Never	Cervical cancer incidence	2	<1%
Appleby 2007	COCP (current user)	Per year of use	Invasive cervical cancer incidence	3	<1%
Appleby 2007	COCP (1-9ys since last use)	Per year of use	Invasive cervical cancer incidence	3	<1%
Appleby 2007	COCP (>10ys since last use)	Per year of use	Invasive cervical cancer incidence	3	<1%
Lee_2016	Environmental tobacco smoke	Yes vs no	Cervical cancer incidence	7	<1%
Wang 2020	GDM	Yes vs no	Cervical cancer incidence	3	<1%
WCRF CUP 2018	Height	Per 5 cm increase	Cervical cancer incidence	4	<1%
Looker 2018	HIV	HIV+, CD4>200 vs HIV-	HPV incidence	5	17%
Looker 2018	HIV	HIV+, CD4</=200 vs HIV-	HPV incidence	3	12%
Looker 2018	HIV	HIV+, CD4>200 vs HIV-	HPV incidence	4	14%
Looker 2018	HIV	HIV+, CD4</=200 vs HIV-	HPV incidence	2	<1%
Looker 2018	HIV	HIV+, CD4>200 vs HIV-	HPV clearance	4	<1%
Looker 2018	HIV	HIV+, CD4</=200 vs HIV-	HPV clearance	2	<1%
Looker 2018	HIV	HIV+ vs HIV-	HPV incidence	11	26%
Looker 2018	HIV	HIV+ vs HIV-	HPV clearance	15	19%
Grulich_2007	HIV	HIV+ vs HIV-	Cervical cancer incidence	6	7%
Liu 2018	HIV	HIV+ vs HIV-	CIN regression	2	7%
Looker 2018	HIV	HIV+ vs HIV-	HPV incidence	15	20%
Looker 2018	HIV	HIV+ vs HIV-	HPV clearance	11	17%
Debeaudrap 2019	HIV	HIV+ vs HIV-	CIN persistence	9	22
Liu 2018	HIV	HIV+ vs HIV-	HPV incidence	3	12%

Liu 2018	HIV	HIV+ vs HIV-	HPV incidence	2	8%
Liu_2018	HIV	HIV+ vs HIV-	HPV incidence	2	8%
Liu 2018	HIV	HIV+ vs HIV-	HPV incidence	2	3%
Liu 2018	HIV	HIV+ vs HIV-	HPV clearance	2	7%
Liu 2018	HIV	HIV+ vs HIV-	HPV clearance	2	7%
Liu 2018	HIV	HIV+ vs HIV-	HPV clearance	3	10%
Liu 2018	HIV	HIV+ vs HIV-	HPV clearance	3	1%
Liu 2018	HIV	HIV+ vs HIV-	HPV clearance	2	8%
Liu 2018	HIV	HIV+ vs HIV-	CIN incidence	2	8%
Looker 2018	HIV	HIV+ vs HIV-	HPV clearance	9	13%
Debeaudrap 2019	HIV	HIV+ vs HIV-	CIN persistence	15	29
Liu 2018	HIV	HIV+ vs HIV-	HPV clearance	2	<1%
Looker 2018	HIV	HIV+ vs HIV-	HPV clearance	7	<1%
Liu 2018	HIV+	CD4 <200 VS >500	HPV clearance	2	7%
Liu 2018	HIV+	CD4 200-500 VS >500	HPV clearance	2	4%
Kelly 2018	HIV+ on treatment	On ART vs no ART	CIN regression	10	16%
Kelly 2018	HIV+ on treatment	On ART vs no ART	CIN incidence	11	3%
Kelly 2018	HIV+ on treatment	On ART vs no ART	CIN progression	10	14%
Kelly 2018	HIV+ on treatment	On ART vs no ART	Invasive cervical cancer incidence	2	6%
Liu 2018	HIV+ on treatment	On ART vs no ART	CIN incidence	3	12%
Kelly 2018	HIV+ on treatment	On ART vs no ART	HPV prevalence	19	<1%
Kelly 2018	HIV+ on treatment	On ART vs no ART	Cervical cancer prevalence	7	<1%
Liu 2018	HIV+ on treatment	On ART vs no ART	CIN incidence	2	<1%
Liu 2018	HIV+ on treatment	On ART vs no ART	CIN regression	2	<1%
Allegreti_2015	IBD on immunosuppressive meds	Yes vs healthy controls	Cervical cancer incidence	5	16%

Li 2013	IVF	Yes vs no	Cervical cancer incidence	4	<1%
Appleby 2006	Parity	Per increase of 1 FTP	CIN incidence	5	<1%
Liu 2014	Pregnancy	Pregnant vs non pregnant	HPV incidence	14	<2%
Helm 2013	Retinoid use	Yes vs no	CIN regression	3	<1%
Helm 2013	Retinoid use	Yes vs no	CIN regression	2	<1%
Helm 2013	Retinoid use	Yes vs no	CIN regression	2	<1%
Simon 2015	Rheumatoid arthritis	Yes vs general population	Cervical cancer incidence	15	<1%
Liu 2015	Sexual partners	Multiple vs few partners	CIN incidence	2	<1%
Kaderli 2014	Smoking	Yes vs no	HPV incidence	10	16%
Appleby 2005	Smoking	Current vs Never	Cervical cancer incidence	4	16%
Appleby 2005	Smoking	Past vs never smoker	Cervical cancer incidence	4	3%
Kaderli 2014	Smoking	Yes vs no	HPV prevalence	5	<1%
Grulich_2007	Transplant recipient	Yes vs no	Cervical cancer incidence	3	9%
Brusselaers 2019	Vaginal dysbiosis	Yes vs no	HPV persistence	7	3%
Tamarelle 2018	VMB	LL-VMB vs HL-VMB	HPV incidence	4	9%
Tamarelle 2018	VMB	LL-VMB vs HL-VMB	HPV incidence	2	<1%
Brusselaers 2019	Vaginal dysbiosis	Yes vs no	CIN incidence	9	16%
Brusselaers 2019	Vaginal dysbiosis	Yes vs no	HPV incidence	4	15%

Abbreviations FTP: full term pregnancy; CIN: cervical intraepithelial neoplasia; HPV: human papilloma virus; LP: lactobacillus predominant; LIP: Lactobacillus iners predominant; LCP: Lactobacillus crispatus predominant; CSTs: community state types; Chalmydia tr: chlamydia trachomatis; COCP: combined oral contraceptive pill; ys: years; GDM: gestational diabetes mellitus; HIV: human immunosufficiency virus; ART: antiretroviral treatment; IBD: inflammatory bowel disease; meds: medications; IVF: in vitro fertilization; VMB: vaginal microbime; LL-VMB: Low lactobacillus vaginal microbiome; HL-VMB: high lactobacillus vaginal microbiome.
Key: [a] Number of studies
