# Supplementary material for: Risk factors for human papillomavirus infection, cervical intraepithelial neoplasia and cervical cancer: an umbrella review and follow-up Mendelian randomisation studies
Source: BMC Med. 2023 Jul 27;21:274. doi: 10.1186/s12916-023-02965-w (PMC10375747; doi:10.1186/s12916-023-02965-w)
Supplement: Supplementary file 8 — Additional file 8: Supplementary Table 7. Details of evidence grading for meta-analyses of risk factors for HPV, pre-invasive and invasive cervical cancer incidence, progression, regression, or mortality - all study types included (sensitivity analysis). [file 12916_2023_2965_MOESM8_ESM.pdf]

Table S7: Details of evidence grading for meta-analyses of risk factors for HPV, pre-invasive and invasive cervical cancer incidence, progression, regression, or mortality - all study types included (sensitivity analysis)

Exposure	Exposure contrast	Outcome	N[a]	Sample size Cases/ Cohort[b]	Largest Study[c]	Random effects Summary RR (95% CI)[d]	Random P-value[e]	95% Prediction interval	Egger's P[f]	I^2 (%)	Excess significance[g]	
											O/E[h]	P-value[i]
**Strong evidence**												
**Sexual health**												
Chlamydia trachomatis infection	Yes vs no	Cervical cancer incidence (CIN1+)	18	3392/7365	2.44 (2.06-2.89)	2.19 (1.74-2.74)	1.03E-11	1.18-4.06	0.34	47	9/13.0	NP
Chlamydia trachomatis infection	Yes vs no	Cervical cancer incidence (SCC)	11	3198/8118	2.55 (2.15-3.03)	2.09 (1.79-2.44)	6.87E-21	1.48-2.95	0.27	32	5/9.6	NP
Chlamydia tr coinfection with HPV	Yes vs no	Cervical cancer incidence (CIN1+)	6	1086/4116	3.23 (2.39-4.35)	4.37 (2.75-6.96)	4.59E-10	1.31-14.66	0.35	44	4/5.2	NP
Chlamydia trachomatis infection	Yes vs no	HR HPV incidence	15	5049/22082	1.84 (1.36-2.47)	2.32 (2.02-2.65)	3.45E-34	2.00-2.69	0.21	0	11/13.4	NP
Number of sexual partners	Multiple vs few	CIN incidence	26	5638/16346	1.94 (1.47-2.41)	1.97 (1.80-2.15)	<1E-100	1.79-2.16	0.43	0	14/22.6	NP
Trichomonas vaginalis infection	Yes vs no	Cervical cancer incidence (CIN1+)	15	7715/75168	1.67 (1.23-2.28)	2.09 (1.69-2.60)	2.30E-11	1.22-3.60	0.93	34	7/9.5	NP
**Contraception**												
Oral contraceptive, medium and long duration >5 years	<8 years since last use vs never	Cervical cancer incidence (CIN3+)	4	5839/20573	2.10 (1.80-2.40)	2.13 (1.87-2.42)	4.37E-31	1.59-2.86	0.45	1	4/4.0	NP

**Immunocompromise**												
HIV infection	HIV+ vs HIV -	HR HPV incidence	11	2323/6345	2.35 (2.03-2.72)	2.20 (1.90-2.54)	3.01E-26	1.63-2.97	0.77	22	10/9.7	0.77
IBD on immunosuppression	Yes vs healthy controls	Cervical cancer incidence (HSIL+)	8	30671/2418502	1.35 (1.28-1.43)	1.34 (1.23-1.46)	5.98E-11	1.12-1.61	0.36	34	4/5.0	NP
**Vitamin supplementation**												
Vitamin C intake	Highest vs lowest	Progression to dysplasia and CIN	9	27405/433341	1.44 (1.22-1.71)	1.59 (1.40-1.82)	5.34E-12	1.20-2.12	0.63	26	5/6.8	NP
Vegetable intake	Highest vs lowest	Cervical cancer incidence	11	2421/32719	0.6 (0.47-0.76)	0.57(0.5-0.66)	1.35E-15	0.49-0.67	0.36	0	4/8.98	NP
**Highly suggestive evidence**												
**Sexual health**												
Chlamydia trachomatis infection	Yes vs no	HPV incidence	40	15642/59248	1.20 (1.09-1.32)	2.12 (1.80-2.49)	3.95E-19	0.90-5.00	<0.01	83	28/11.4	<0.01
Number of sexual partners	Multiple partners vs few partners	Cervical cancer incidence	24	5878/16319	1.56 (1.28-1.84)	1.99 (1.67-2.38)	1.96E-14	1.11-3.58	0.32	54	13/16.9	NP
**Immunocompromise**												
HIV infection	HIV+ vs HIV -	Clearance of HPV	15	2977/4471	0.67 (0.56-0.81)	0.53 (0.43-0.64)	4.32E-10	0.26-1.06	0.37	73	8/7.4	0.74
HIV infection	HIV+ vs HIV -	Treatment failure for any grade lesions	18	1431/3434	3.20 (2.40-4.30)	5.34 (4.21-6.77)	<1E-100	2.62-10.85	<0.01	44	17/16.1	0.49
HIV infection	HIV+ vs HIV -	Cervical cancer incidence	6	1160/12269	5.2 (3.81-6.93)	5.81(3-11)	2.34E-07	0.67-50.17	0.78	86	4/4.08	NP
HIV infection	HIV+ with CD4>200 vs HIV-	HPV incidence	5	1151/2335	3.61(3.01-4.32)	3.09(2.17-4.4)	3.75E-10	0.91-10.55	0.71	83	5/5	0.96
**Smoking**												
Smoking	Ever vs never	Cervical cancer incidence (CIN3+)	35	4749/11844	1.60 (1.40-1.90)	1.78 (1.55-2.04)	2.15E-16	0.85-3.71	0.24	83	22/17.8	0.16
Smoking	Current vs never	CIN incidence	27	2018/7088	2.30 (1.70-3.00)	2.21 (1.89-2.58)	2.88E-23	1.19-4.09	0.60	56	19/17.0	0.43
Smoking	Current vs never	Cervical cancer incidence (CIN3+)	19	10814/28619	2.26 (1.88-2.72)	1.69 (1.40-2.02)	2.10E-08	0.81-3.51	0.73	78	11/18.8	NP
**Contraception**												

Oral contraceptives	Long duration >10 years vs never	Invasive cervical cancer incidence	13	5615/23166	2.20 (1.90-2.70)	2.14 (1.66-2.76)	3.39E-09	0.96-4.80	0.99	65	8/11.5	NP
COC current users	Risk per year of use	Cervical cancer incidence	18	1105/3546	1.09 (1.07-1.11)	1.06 (1.04-1.08)	2.50E-10	1.01-1.11	0.057	32	6/1.1	<0.01
**Pregnancy**												
Age in pregnancy	Per 1-year decrease in age at FTP	Cervical cancer incidence	17	9871/32095	1.07 (1.05-1.09)	1.07 (1.05-1.08)	1.92E-19	1.03-1.10	0.98	25	7/1.9	<0.01
Parity	Per increase of 1 FTP	Cervical cancer incidence	17	9764/25297	1.08 (1.05-1.10)	1.11 (1.09-1.14)	1.22E-16	1.04-1.19	0.64	41	11/2.1	<0.01
**Vitamin supplementation**												
Vitamin C intake	By increased intake of 50mg/d	Cervical cancer incidence (CIN1+)	9	2405/7039	0.94 (0.91-0.98)	0.91(0.89-0.94)	1.09E-09	0.88-0.95	0.01	3	5/0.7	<0.01
Serum selenium levels*	Highest vs lowest	Cervical cancer incidence	21	1372/2602	-1.08 (-1.42- -0.75)	-4.84 (-5.99- -3.68)	2.14E-16	NA	<0.01	99	2-13.4	<0.01
**Suggestive evidence**												
**Sexual health**												
Bacterial Vaginosis	Yes vs no	CIN incidence	20	110722/1544804	1.19 (1.13-1.26)	1.50 (1.24-1.82)	3.40E-05	0.73-3.07	0.27	89	11/6.0	0.01
Candida albicans	Yes vs no	HPV incidence	5	1380/8860	0.48(0.33-0.71)	0.63(0.48-0.82)	9.00E-04	0.39-1.02	0.68	3	1/4.2	NP
Vaginal douching	Yes vs no	Cervical cancer incidence (CIN3+)	12	1896/5805	1.40 (1.10-1.90)	1.45 (1.19-1.77)	2.50E-04	0.81-2.61	0.12	51	5/4.5	0.75
Vaginal douching	>/= 1 time/week vs less	Cervical cancer incidence (CIN3+)	5	1928/6290	1.50 (1.00-2.40)	2.06 (1.34-3.15)	8.80E-04	0.51-8.25	0.13	63	3/4.3	NP
Vaginal dysbiosis	Yes vs no	HPV incidence	4	1926/3354	1.24 (1.04-1.47)	1.33 (1.18-1.50)	2.24E-06	1.03-1.73	0.31	0	3/2.0	0.30
VMB, less than 40 years old	LL-VMB vs HL-VMB	HPV incidence	7	1772/5545	2.11 (1.54-2.91)	1.69 (1.31-2.18)	5.30E-05	0.85-3.37	0.54	51	4/6.3	NP
**Immunocompromise**												
HIV infection	Yes vs no	Cervical cancer incidence	9	1184/141492	5.20 (3.81-6.93)	3.98 (2.24-7.05)	2.32E-06	0.59-26.73	0.30	86	5/6.1	NP
HIV infection	HIV+ vs HIV -	Clearance of prevalent and newly detected HPV (any)	3	1007/2663	0.46 (0.39-0.54)	0.47 (0.33-0.68)	5.80E-05	0.01-39.10	0.65	87	3/2.98	0.90
HIV infection	HIV+ vs HIV -	LSIL regression	2	1968/2586	0.69 (0.57-0.84)	0.67 (0.56-0.81)	3.50E-05	NA	NA	0	1/1.9	NP

HIV infection	HIV+ vs HIV -	HPV incidence	15	1722/6620	1.35 (0.99-1.84)	1.73 (1.39-2.16)	1.01E-06	0.91-3.31	0.57	47	6/3.6	0.16
HIV infection	HIV+ vs HIV -	Clearance of HR HPV	11	3581/6322	0.67 (0.59-0.77)	0.66 (0.55-0.79)	8.03E-06	0.37-1.20	0.40	77	9/6.0	0.07
HIV+ treatment	ART vs no ART	CIN regression (SIL)	10	3074/4879	1.30 (1.00-1.70)	1.62 (1.32-1.99)	4.41E-06	0.93-2.83	0.38	49	6/5.3	0.67
HIV+ treatment	ART vs no ART	Cervical cancer incidence	3	2360/409519	0.50 (0.29-0.87)	0.43 (0.26-0.71)	8.80E-04	0.02-10.57	0.41	0	2/2.2	NP
**Contraception**												
IUD device use	Any vs no	Invasive cervical cancer incidence	16	4945/12482	0.89 (0.73-1.08)	0.64 (0.53-0.77)	1.91E-06	0.38-1.07	0.03	42	5/2.2	0.04
IUD device use	Use vs no use	Cervical cancer incidence	8	2205/4419	0.45 (0.30-0.67)	0.49 (0.34-0.70)	9.60E-05	0.21-1.13	0.67	34	3/7.6	NP
Oral contraceptives	Medium duration 5-9 years vs never	Invasive cervical cancer incidence	21	7930/30007	1.70 (1.50-2.00)	1.50 (1.27-1.77)	1.15E-06	0.84-2.69	0.22	58	9/16.0	NP
**Smoking**												
Environmental tobacco smoke	Yes vs no	Cervical cancer incidence (CIN2+)	17	2039/79240	1.30 (1.07-1.59)	1.60 (1.32-1.93)	1.90E-06	0.84-3.03	0.11	59	7/5.3	0.37
Passive smoking	Yes vs no	Cervical cancer incidence (CIN1+)	16	2230/7284	1.03 (0.99-1.07)	1.68 (1.32-2.15)	2.80E-05	0.67-4.21	<0.01	82	9/0.8	<0.01
Smoking	Current vs never	Cervical cancer incidence (CIN3+)	24	2702/9258	1.10 (0.90-1.40)	2.04 (1.70-2.44)	8.5E-15	0.88-4.75	0.67	84	16/2.6	<0.01
Smoking	Ever vs never	CIN incidence	25	2243/8384	1.30 (1.00-1.70)	1.96 (1.69-2.27)	2.65E-19	1.10-3.49	0.05	57	19/4.6	<0.01
Smoking	Past smoker vs never	Cervical cancer incidence (CIN3+)	19	10814/28619	1.29 (1.04-1.60)	1.21 (1.09-1.34)	3.20E-04	1.08-1.35	0.80	0	3/11.1	NP
Smoking	Yes vs no	HPV incidence	10	9442/18745	1.20 (1.00-1.30)	1.37 (1.21-1.56)	1.04E-06	1.06-1.77	0.48	22	4/4.5	NP
**Vitamin supplementation**												
Vitamin A intake	Highest vs lowest	Invasive cervical cancer incidence	22	3418/10478	0.95 (0.74-1.22)	0.59 (0.49-0.72)	1.51E-07	0.25-1.39	0.38	78	12/1.3	<0.01
Vitamin C intake	Highest vs lowest	Cervical cancer incidence (CIN1+)	12	3711/307410	0.98 (0.81-1.19)	0.58(0.46-0.74)	7.86E-06	0.27-1.26	0.02	70	6/0.6	<0.01
Vitamin E supplement intake	Highest vs lowest	Cervical cancer incidence	15	3815/10473	0.35 (0.28-0.44)	0.58 (0.47-0.72)	1.15E-06	0.25-1.37	0.51	83	11/14.9	NP
Vitamin E supplement intake	Highest vs lowest	Invasive cervical cancer incidence	9	1321/4177	0.48 (0.38-0.61)	0.53 (0.39-0.73)	7.30E-05	0.19-1.50	0.77	78	6/8.0	NP

Vitamin E supplement intake	Highest vs lowest	CIN incidence	17	2494/6296	0.66 (0.51-0.87)	0.56 (0.45-0.71)	1.06E-06	0.22-1.41	0.36	76	11/8.6	0.24
Fruit intake	Highest vs lowest	Cervical cancer incidence	6	1423/32346	0.76 (0.62-0.93)	0.79 (0.67-0.92)	3.00E-03	0.63-0.99	0.65	0	1/3.04	NP
Retinol intake	Highest vs lowest	Cervical cancer incidence	19	2925/9034	0.95 (0.74-1.22)	0.61 (0.49-0.76)	8.40E-06	0.25-1.50	0.74	79	10/1.1	<0.01
**Weak evidence**												
**Immunocompromise**												
EBV infection	Yes vs no	CIN1 incidence	6	52/193	2.69 (1.22-5.91)	2.32 (1.18-4.54)	0.01	0.58-9.19	0.95	18	2/1.3	0.50
EBV infection	Yes vs no	CIN2/3 incidence	7	79/226	6.30 (2.94-13.54)	2.35 (1.06-5.22)	0.04	0.24-23.47	0.11	59	3/3.6	NP
EBV infection	Yes vs no	Cervical cancer incidence	13	166/354	0.20 (0.08-0.48)	4.05 (1.78-9.24)	8.70E-04	0.20-80.35	0.08	78	10/5.9	0.02
HIV infection	HIV+ vs HIV -	Clearance of HPV 16	9	2283/2681	0.56 (0.39-0.81)	0.67 (0.50-0.90)	7.30E-03	0.28-1.61	0.25	60	4/5.5	NP
HIV infection	HIV+ vs HIV -	Treatment failure for high grade lesions	10	349/2339	3.50 (2.00-6.10)	2.66 (2.00-3.54)	2.14E-11	1.90-3.73	0.36	0	4/8.5	NP
HIV infection	HIV+ vs HIV -	LSIL incidence	2	362/2382	4.00 (2.61-6.13)	3.73 (2.62-5.32)	3.46E-13	NA	NA	0	2/2.0	NP
HIV infection	HIV+ vs HIV -	HPV incidence	3	528/1166	2.98 (2.07-4.29)	2.64 (2.04-3.42)	1.13E-13	0.50-13.94	0.96	0	2/3.0	NP
HIV infection	HIV+ vs HIV -	HR HPV incidence	2	263/947	2.30 (1.57-3.37)	2.35 (1.64-3.37)	3.62E-06	NA	NA	0	1/1.8	NP
HIV infection	HIV+ vs HIV -	HPV 16 incidence	2	91/973	3.09 (1.39-6.86)	3.05 (1.71-5.43)	1.50E-04	NA	NA	0	2/1.9	0.74
HIV infection	HIV+ vs HIV -	HPV 18 incidence	2	54/996	3.19 (1.17-8.70)	2.55 (1.17-5.60)	0.02	NA	NA	0	1/1.7	NP
HIV infection	HIV+ vs HIV -	Clearance of newly detected HPV	2	569/3593	0.67 (0.56-0.81)	0.74 (0.59-0.92)	8.20E-03	NA	NA	47	1/2.0	NP
HIV infection	HIV+ vs HIV -	Clearance of prevalent and newly detected HPV (Any HPV)	2	424/820	0.46 (0.34-0.62)	0.34 (0.16-0.75)	7.20E-03	NA	NA	63	2/1.8	0.66
HIV infection	HIV+ vs HIV -	Clearance of prevalent and newly detected HPV (HR HPV)	2	617/1375	0.60 (0.47-0.76)	0.53 (0.37-0.75)	3.86E-04	NA	NA	47	2/1.4	0.35
HIV infection	HIV+ vs HIV -	Clearance of prevalent and newly detected HPV (HR HPV)	3	675/960	0.75 (0.59-0.96)	0.58 (0.39-0.87)	7.80E-03	0.01-44.81	0.50	61	2/0.9	0.18
HIV infection	HIV+ vs HIV -	Clearance of prevalent and newly detected HPV (HPV 16)	3	485/509	0.57 (0.41-0.79)	0.64 (0.44-0.93)	0.02	0.02-10.10	0.37	30	1/2.2	NP

HIV infection	HIV+ vs HIV -	Clearance of prevalent and newly detected HPV (HPV 18)	2	348/1282	0.50 (0.31-0.81)	0.48 (0.32-0.72)	4.30E-04	NA	NA	0	2/1.8	0.59
HIV+	ART vs no ART	HR HPV prevalence	20	4163/4592	0.83 (0.74-0.94)	0.82 (0.68-0.99)	0.04	0.41-1.64	0.37	71	4/3.4	0.72
HIV+	ART vs no ART	CIN incidence	12	2190/6100	0.62 (0.42-0.91)	0.70 (0.55-0.90)	4.30E-03	0.39-1.28	0.43	34	4/7.6	NP
HIV+	ART vs no ART	SIL progression	10	3493/5075	0.66 (0.54-0.81)	0.74 (0.61-0.90)	2.60E-03	0.42-1.31	0.13	60	5/6.9	NP
HIV+	ART vs no SRT	LSIL incidence	3	522/1082	0.66 (0.47-0.92)	0.65 (0.52-0.82)	2.80E-04	0.15-2.91	0.65	0	2/1.8	0.79
HIV+, CD4 count	CD4 <200 VS >500	Clearance of HPV	2	812/1406	0.47 (0.38-0.59)	0.46 (0.37-0.57)	1.11E-12	NA	NA	0	2/1.99	0.91
HIV+, CD4 count	CD4 200-500 VS >500	Clearance of HPV	2	812/1406	0.76 (0.64-0.90)	0.76 (0.64-0.89)	1.00E-03	NA	NA	0	1/1.2	NP
HIV+, CD4 count	CD4 <200 VS >200	Clearance of HPV	1	764/1320	0.57 (0.39-0.83)	0.57 (0.39-0.83)	3.50E-03	NA	NA	100	1/0.9	0.73
HIV infection	HIV+ with CD4>200 vs HIV-	HPV incidence	3	703/1212	5.19(4.3-6.26)	5.76(3.65-9.1)	4.73E-14	0.04-844.24	0.75	62	3/3	0.99
HIV infection	HIV+ with CD4</=200 vs HIV-	HPV incidence	4	327/758	1.41(0.89-2.24)	1.94(1.45-2.6)	1.80E-03	0.36-11.36	0.4	55	2/1.12	0.30
SLE	Yes vs healthy controls	HSIL incidence	7	78/11814	25.45 (9.53-67.98)	9.01 (4.19-19.41)	1.92E-08	1.80-45.26	0.25	23	3/6.8	NP
Rheumatoid arthritis	Yes vs general population	Cervical cancer incidence	15	297/297590	0.86 (0.84-0.89)	0.85 (0.73-1.00)	0.05	0.54-1.34	0.97	63	3/1.0	0.04
Transplant recipient	Yes vs no	Cervical cancer incidence	3	24/188000	2.5(1.33-4.2)	2.31(1.4-3.3)	7.00E-04	0.12-36.68	0.39	0	1/0	0.89
**Sexual health**												
Bacterial Vaginosis	Yes vs no	HPV infection	12	7921/14883	1.28 (1.16-1.41)	1.43 (1.11-1.84)	5.20E-03	0.69-2.98	0.39	60	3/4.0	NP
Cervicovaginal lactobacilli	LP CSTs vs non LP CST	HR HPV incidence	9	415/890	0.78 (0.45-1.36)	0.64 (0.47-0.88)	5.40E-03	0.40-1.01	0.53	5	1/0.9	0.86
Cervicovaginal lactobacilli	LP CSTs vs non LP CST	CIN incidence	6	264/543	0.70 (0.35-1.38)	0.56 (0.35-0.90)	0.02	0.29-1.09	0.38	0	0/0.7	NP

Cervicovaginal lactobacilli	LP CSTs vs non LP CST	Cervical cancer incidence	3	23/111	0.18 (0.04-0.78)	0.14 (0.04-0.45)	8.90E-04	0-250.3	0.33	0	2/1.8	0.80
Cervicovaginal lactobacilli	LIP CST III vs non LIP CST III	Cervical cancer incidence	2	13/50	0.13 (0.01-2.75)	0.13 (0.02-0.95)	0.04	NA	NA	0	0/1.3	NP
Cervicovaginal lactobacilli	LCP CST I vs non LCP CST I	HR HPV incidence	8	329/762	0.58 (0.24-1.44)	0.53 (0.31-0.90)	0.02	0.25-1.11	0.35	3	1/2.1	NP
Cervicovaginal lactobacilli	LCP CST I vs non LCP CST I	CIN incidence	5	226/454	0.44 (0.19-1.03)	0.51 (0.29-0.91)	0.02	0.20-1.29	0.55	0	0/1.7	NP
Chlamydia trachomatis infection	Yes vs no	Cervical cancer incidence (CIN3+)	3	832/3473	2.21 (1.84-2.65)	2.21 (1.62-3.03)	6.43E-07	0.09-53.54	0.96	46	2/2.9	NP
Chlamydia trachomatis infection	Yes vs no	Cervical cancer incidence (CIN1+)	11	3321/9111	1.90 (0.82-4.29)	1.76 (1.03-3.01)	0.04	0.95-3.27	0.17	0	0/9.1	NP
Chlamydia tr coinfection with HPV	Yes vs no	Cervical cancer incidence (CIN1+)	6	332/4686	3.23 (2.39-4.35)	4.37 (2.75-6.96)	4.59E-10	1.31-14.66	0.35	44	4/4.5	NP
Chlamydia trachomatis infection	Yes vs no	Cervical cancer incidence (ACC)	4	262/1848	1.46 (0.96-2.23)	1.60 (1.19-2.14)	1.70E-03	0.84-3.04	0.12	0	2/1.3	0.43
HSV type 2 infection	Yes vs no	Cervical cancer incidence (CIN3+)	20	3337/21251	1.10 (0.80-1.40)	1.21 (1.04-1.41)	0.02	1.03-1.43	0.43	0	1/1.7	NP
M.genitalium infection	Yes vs no	HR HPV incidence	5	1355/3337	1.57 (1.03-2.40)	1.55 (1.00-2.40)	0.05	0.56-4.26	0.66	19	1/3.9	NP
M.hominis infection	Yes vs no	Cervical cancer incidence (CIN1+)	6	1412/2037	1.34 (0.84-2.16)	1.48 (1.10-1.98)	9.10E-03	0.98-2.24	0.97	0	1/1.4	NP
U.urealyticum infection	Yes vs no	HPV incidence	8	2388/7071	1.38 (1.21-1.56)	1.57 (1.05-2.34)	0.03	0.48-5.09	0.42	80	5/3.5	0.28
U.parvum infection	Yes vs no	HPV incidence	3	187/692	2.54 (1.48-4.38)	3.03 (1.75-5.26)	8.20E-05	0.01-1011	0.88	55	2/2.7	NP
U.urealyticum infection	Yes vs no	HR HPV incidence	4	2148/6197	1.34 (1.17-1.54)	1.37 (1.05-1.80)	0.02	0.50-3.79	0.56	54	2/2.3	NP
U.urealyticum infection	Yes vs no	Cervical cancer incidence (CIN1+)	9	1865/2731	1.33 (0.90-1.98)	1.51 (1.14-2.01)	4.10E-03	0.77-2.97	0.49	36	4/2.0	0.11
VMB, all ages	LL-VMB vs HL-VMB	HPV incidence	13	4481/13118	2.42 (1.92-3.05)	1.53 (1.16-2.01)	2.30E-03	0.62-3.78	0.44	73	2/10.8	NP
Vaginal dysbiosis	Yes vs no	HPV incidence	7	1719/2992	1.03 (0.97-1.09)	1.14 (1.01-1.28)	0.03	0.86-1.50	0.23	44	3/0.4	<0.01

Vaginal douching	>/= 20 years vs less	Cervical cancer incidence (CIN3+)	3	1244/4494	1.95 (1.19-3.03)	1.79 (1.23-2.61)	2.20E-03	0.12-25.87	0.27	6	2/3.0	NP
**Vitamin supplementation**												
Beta carotene	Highest intake (or serum level) vs lowest	Cervical cancer incidence (CIN1+)	9	1965/6047	0.89 (0.60-1.30)	0.67 (0.51-0.88)	3.80E-03	0.35-1.26	0.45	32	2/1.1	0.35
Carotenoid's intake	Highest vs lowest	Invasive cervical cancer incidence	8	980/2275	0.21 (0.15-0.30)	0.58 (0.37-0.92)	0.02	0.12-2.79	0.20	84	3/8.0	NP
Folate	Highest intake (or serum level) vs lowest)	Cervical cancer incidence (CIN1+)	9	1850/5329	1.05 (0.70-1.60)	0.60 (0.41-0.88)	8.40E-03	0.19-1.83	0.09	60	3/0.5	<0.01
Lycopene	Highest intake (or serum level) vs lowest)	Cervical cancer incidence (CIN1+)	8	694/2222	0.65 (0.44-0.98)	0.53 (0.37-0.75)	4.00E-04	0.28-1.02	0.04	4	3/2.7	0.80
Serum selenium levels	Highest vs lowest	Cervical cancer incidence	5	353/1206	0.58 (0.37-0.91)	0.55 (0.42-0.73)	2.20E-05	0.35-0.86	0.21	0	3/2.7	0.77
Serum iron levels	Highest vs lowest	Cervical cancer incidence	9	454/880	-0.51(-0.8- -0.22)	-1.45 (-1.98- -0.91)	1.02E-0.7	NA	0.11	93		
Serum zinc levels*	Highest vs lowest	Cervical cancer incidence	12	591/1544	-1.16 (-1.45- -0.87)	-5.34 (-6.84- -3.84)	3.19E-12	NA	<0.01	99	11/10.15	0.50
Serum copper levels*	Highest vs lowest	Cervical cancer incidence	14	747/1761	0.86 (0.58-1.14)	1.35 (0.99-2.59)	0.03	NA	0.72	98	13/12.86	0.89
Vitamin A levels	Highest vs lowest	Invasive cervical cancer incidence	9	761/2320	0.79 (0.54-1.14)	0.60 (0.41-0.89)	0.01	0.18-2.06	0.25	73	4/1.5	0.02
**Pregnancy**												
Pregnancy	Pregnant vs not pregnant	HPV incidence	14	1474/8044	0.87 (0.66-1.14)	1.42 (1.03-1.96)	0.03	0.42-4.80	0.93	82	6/1.5	<0.01
Age in pregnancy	Per one-year decrease in age at FTP	CIN3 incidence	15	3618/20466	1.04 (1.02-1.07)	1.04 (1.01-1.07)	0.02	0.94-1.14	0.86	59	4/0.9	<0.01
**Contraception**												
Condom use	Frequently vs rarely	Cervical cancer incidence	5	1648/8150	0.70 (0.50-1.20)	0.61 (0.38-0.98)	0.04	0.13-2.81	0.25	62	1/3.5	NP
Condom use	Ever vs never	CIN2/3 incidence	7	1491/5198	0.60 (0.40-0.90)	0.71 (0.51-0.97)	0.03	0.30-1.67	0.11	50	3/5.5	NP
OC, short duration <5 ys	<8 years since last use vs never	Cervical cancer incidence (CIN3+)	4	5660/20174	1.30 (1.10-1.50)	1.57 (1.16-2.14)	3.80E-03	0.47-5.30	0.30	61	2/2.5	NP

OC, medium and long duration >5 ys	>8 years since last use vs never	Cervical cancer incidence (CIN3+)	4	5520/20091	1.40 (1.00-1.90)	1.50 (1.04-2.15)	0.03	0.53-4.22	0.62	13	2/2.7	NP
OC, long duration >10 years	Yes vs never	Cervical cancer incidence (CIN2+)	3	1279/1415	1.50 (0.80-2.90)	2.55 (1.15-5.64)	0.02	0-9195	0.88	56	1/1.2	NP
**Smoking**												
Household Air Pollution	Yes vs no	CIN incidence	4	663/2410	3.46 (1.08-11.10)	6.46 (3.12-13.35)	4.85E-07	1.31-31.81	0.38	0	3/4.0	NP
Smoking	Yes vs no	HPV prevalence	5	4451/6402	1.20 (1.00-1.40)	1.24 (1.03-1.50)	0.03	0.66-2.31	0.46	66	3/1.0	0.02
Smoking	Ever vs never	CIN incidence	2	211/239	2.22 (0.49-10.10)	3.72 (1.23-11.24)	0.02	NA	NA	0	1/0.7	0.61
Smoking	Current vs never	Cervical cancer incidence (CIN3+)	19	798/2075	1.68(1.17-2.41)	1.85(1.47-2.33)	2.22E-0.7	1.11-3.09	0.84	0.71	2/3.36	NP
**Anthropometric indices**												
BMI	Highest vs lowest	Cervical cancer mortality	4	420/3144041	1.15 (0.79-1.70)	1.82 (1.07-3.10)	0.03	0.22-15.20	0.63	65	2/0.5	0.02
**No significant association**												
**Immunocompromise**												
HIV infection	HIV+ vs HIV -	Clearance of HPV 18	7	2170/2353	0.91 (0.62-1.33)	0.77 (0.52-1.15)	0.21	0.24-2.48	1.00	61	3/0.6	<0.01
HIV infection	HIV+ vs HIV -	Clearance of prevalent and newly detected HPV 16	2	105/1389	1.61 (0.74-3.51)	0.82 (0.21-3.20)	0.77	NA	NA	81	1/1.1	NP
HIV infection	HIV+ vs HIV -	Clearance of prevalent and newly detected HPV 18	2	74/1389	1.22 (0.85-1.76)	1.04 (0.58-1.86)	0.89	NA	NA	31	0/1.1	NP
HIV+	CD4 <350 VS >350	Clearance of HPV	1	319/486	0.77 (0.53-1.10)	0.77 (0.53-1.11)	0.16	NA	NA	100	0/1.0	NP
HIV+	ART vs no ART	LSIL incidence	2	358/896	0.55 (0.34-0.89)	0.70 (0.39-1.25)	0.23	NA	NA	48	1/1.8	NP
HIV+	ART vs no ART	Cervical cancer incidence (HSIL+)	14	1617/8910	1.01 (0.79-1.30)	0.91 (0.70-1.20)	0.51	0.40-2.08	0.28	57	3/0.7	0.01
HIV+	Yes vs no	LSIL regression	2	325/824	2.61 (1.75-3.89)	1.11 (0.16-7.61)	0.91	NA	NA	84	1/1.7	NP
HIV infection	HIV+ with CD4>200 vs HIV-	HR HPV incidence	2	152/334	1.26 (0.77-2.06)	1.3 (0.82-2.070)	0.26	NA	NA	NA	0/0.3	0.60
HIV infection	HIV+ with CD4</=200 vs HIV-	Clearance of HPV	4	299/468	1.49 (1.09-2.04)	1.186 (0.89 -1.58)	0.24	0.51-2.77	0.137	0.51-2.77	0/1.19	0.84

HIV infection	HIV+ with CD4>200 vs HIV-	Clearance of HR HPV	2	153/214	1.33 (0.92-1.93)	1.18 (0.8-1.74)	0.40	NA	NA	NA	0/0.4	0.50
**Genitourinary health**												
Candida albicans	Yes vs no	CIN incidence	2	444/6459	0.8(0.32-1.97)	0.99(0.48-2.1)	0.99	NA	NA	NA	0/0.72	0.30
Cervicovaginal lactobacilli	LIP CST III vs non LIP CST III	HR HPV incidence	8	329/805	0.85 (0.48-1.51)	0.95 (0.68-1.33)	0.78	0.63-1.45	0.50	0	0/0.6	NP
Cervicovaginal lactobacilli	LIPCST III vs non LIP CST III	CIN incidence	5	226/454	1.25 (0.62-2.53)	1.01 (0.61-1.68)	0.96	0.44-2.31	0.71	0	0/0.4	NP
Cervicovaginal lactobacilli	LCPCST I vs non LCP CST I	Cervical cancer incidence	2	13/50	0.25 (0.02-2.65)	0.16 (0.03-1.00)	0.050	NA	NA	0	0/0.7	NP
M.hominis infection	Yes vs no	HPV incidence	3	154/514	1.52 (0.74-3.14)	1.65 (0.93-2.92)	0.08	0.04-66.38	0.77	0	0/0.8	NP
M.genitalium infection	Yes vs no	Cervical cancer incidence (CIN1+)	5	1243/2415	0.80 (0.37-1.72)	0.80 (0.43-1.51)	0.50	0.19-3.45	0.95	20	0/1.0	NP
**Contraception**												
Condom use	Frequently vs rarely	HPV incidence	6	1144/3292	1.50 (1.10-2.00)	0.94 (0.59-1.49)	0.78	0.21-4.24	0.102	76	2/3.2	NP
Condom use	Ever vs never	CIN incidence	4	1560/6898	1.80 (1.40-2.40)	1.31 (0.93-1.82)	0.12	0.34-5.03	0.57	62	1/3.8	NP
COCP, 2-9 years since last use	Risk per year of use	Cervical cancer incidence	18	1190/4044	1.05 (1.03-1.08)	1.02 (1.00-1.04)	0.060	0.96-1.08	0.21	40	4/1.0	<0.01
COCP, 10+ years since last use	Risk per year of use	Cervical cancer incidence	18	1043/4537	1.00 (0.96-1.04)	0.98 (0.95-1.01)	0.19	0.88-1.09	0.26	58	4/0.9	<0.01
Injectable contraceptives	<5 years of use vs never	Invasive cervical cancer incidence	3	3147/14427	1.10 (0.90-1.30)	0.90 (0.56-1.45)	0.66	0-218.60	0.61	73	1/0.9	0.91
Injectable contraceptives	>5 years of use vs never	Cervical cancer incidence (CIN3+)	2	2504/12372	1.20 (0.90-1.50)	1.49 (0.79-2.80)	0.21	NA	NA	55	1/1.6	NP
IUD device use	Use vs no use	HPV incidence	16	2093/15382	1.09 (0.80-1.47)	0.98 (0.86-1.12)	0.77	0.80-1.20	0.23	6	0/1.3	NP
OC, short duration <5 years	>8 years since last use vs never	Cervical cancer incidence (CIN3+)	4	5647/20369	1.20 (1.00-1.30)	0.98 (0.75-1.27)	0.87	0.36-2.67	<0.01	52	1/1.8	NP
Oral contraceptive	<5 years user vs never	Cervical cancer incidence (CIN3+)	5	1624/2544	0.70 (0.50-1.00)	0.95 (0.69-1.31)	0.75	0.45-2.03	0.09	22	1/1.7	NP
Oral contraceptive	5-9 years user vs never	Cervical cancer incidence (CIN2+)	5	1464/2132	1.20 (0.70-2.20)	1.32 (0.79-2.21)	0.28	0.30-5.93	0.76	49	1/0.6	0.59

Oral contraceptive	Ever vs never	Cervical cancer incidence (CIN3+)	16	7433/15619	0.68 (0.55-0.84)	1.12 (0.90-1.38)	0.31	0.48-2.62	0.35	83	7/10.9	NP
Oral contraceptives	<5 years user vs never	Invasive cervical cancer incidence	20	9149/32642	1.20 (1.10-1.30)	1.15 (1.00-1.32)	0.053	0.71-1.84	0.98	60	4/6.7	NP
**Pregnancy**												
Parity	Per increase of 1 full term pregnancy	CIN3 incidence	15	4620/25024	1.05 (1.01-1.06)	1.08 (1.00-1.16)	0.061	0.83-1.39	0.64	71	4/1.0	<0.01
IVF	Yes vs no	Cervical cancer incidence	4	33735/1420192	0.61 (0.52-0.71)	1.07 (0.45-2.55)	0.87	0.03-34.71	0.29	69	2/1.4	0.56
**Smoking**												
Smoking	Ever vs Never	Cervical cancer incidence	7	1401/1596	1.80 (0.81-4.04)	1.62 (0.91-2.89)	0.10	0.48-5.47	0.89	23	1/2.5	NP
Smoking	Past vs never	Cervical adenocarcinoma incidence	10	1417/16709	0.75 (0.53-1.07)	0.92 (0.75-1.34)	0.45	0.72-1.18	0.48	0	0/4.0	NP
Smoking	Current vs never	Cervical adenocarcinoma incidence	10	1417/15292	0.81 (0.58-1.13)	0.90 (0.72-1.12)	0.34	0.59-1.39	0.60	20	0/2.5	NP
Smoking	Past vs never	Cervical cancer incidence (CIN3+)	19	798/2075	1.83(1.19-2.82)	1.53(1.12-2.1)	0.08	0.73-3.20	0.13	0.73-3.2	1/3.61	NP
**Anthropometric indices**												
Height	Per 5 cm increase	Cervical cancer incidence	4	1182/989044	1.01 (0.92-1.11)	1.02 (0.87-1.20)	0.77	0.57-1.83	0.76	44	0/0.2	NP
BMI	Per 5KG/M2 increase	Cervical cancer incidence	9	5035/9680798	0.98 (0.95-1.02)	1.02 (0.97-1.07)	0.44	0.89-1.17	0.79	72	2/0.5	0.04
BMI	Per 5KG/M2 increase	Cervical cancer incidence	7	4837/7870866	0.98 (0.95-1.02)	1.01 (0.96-1.06)	0.67	0.87-1.17	0.96	68	1/0.4	0.36
BMI	Highest vs lowest	Cervical cancer incidence	7	4837/7870866	1.02 (0.80-1.31)	1.13 (0.93-1.38)	0.23	0.71-1.79	0.94	33	1/0.4	0.35
BMI	Per 5KG/M2 increase o	Cervical cancer mortality	3	307/3032453	1.04 (0.88-1.24)	1.02 (0.83-1.25)	0.84	0.11-9.52	0.86	65	0/0.2	NP
**Medical history**												
GDM	Yes vs no	Cervical cancer incidence	3	63694/1163875	0.9(0.65-1.26)	1.02(0.81-1.28)	0.84	0.23-4.49	0.04	0.04	0/3	NP
**Vitamin supplementation**												

Retinoid use	Yes vs no	Complete or partial regression of CIN2/3 at 3-12 months	3	186/314	0.92 (0.39-2.16)	0.98 (0.56-1.72)	0.95	0.03-36.66	0.16	0	0/0.2	NP
Retinoid use	Yes vs no	Complete regression of CIN2/3 at 9-27 months	2	160/246	0.71 (0.41-1.24)	0.79 (0.51-1.23)	0.29	NA	NA	0	0/0.5	NP
Retinoid use	Yes vs no	Complete regression of CIN2 at 9-27 months	2	116/144	0.50 (0.25-1.02)	0.81 (0.29-2.28)	0.69	NA	NA	69	0/1.1	NP
Vegetable intake	Highest vs lowest intake	Cervical dysplasia	5	1916/303706	0.98(0.77-1.25)	0.79(0.56-1.12)	0.19	0.32-1.95	0.19	31	0/0.27	0.59
Vegetable intake	Highest vs lowest intake	Cervical dysplasia	3	515	1.03(0.55-1.92)	1.14(0.74-1.76)	0.55	0.07-19.02	0.1	0	0/0.16	0.68
Vegetable intake	Highest vs lowest intake	Cervical cancer incidence	3	957/2967	0.97(0.97-0.71)	0.9(0.68-1.2)	0.47	0.15-5.44	0.23	0	0/0.17	0.67
Fruit intake	Highest vs lowest intake	Cervical dysplasia	3	1191/300924	1.06(0.85-1.32)	0.54(0.2-1.41)	0.21	0-5573	0.24	83	1/0.28	0.16
Fruit intake	Highest vs lowest intake	Cervical dysplasia	3	515/1317	0.97(0.93-1.02)	0.89(0.69-1.15)	0.38	0.07-10.64	0.13	21	0/0.16	0.68
Fruit intake	Highest vs lowest intake	Cervical cancer incidence	2	856/2720	0.86(0.61-1.22)	0.87(0.64-1.2)	0.4	NA	NA	NA	0/0.6	0.40

Abbreviations: ART, antiretroviral therapy; BMI, body mass index; CC, cervical cancer; CD4, cluster of differentiation 4; Chalmydia tr: chlamydia trachomatis; CI, confidence interval; CIN: cervical intra-epithelial neoplasia; COC, combined oral contraceptive; CST, community state types; EBV, Epstein-Barr virus; FTP: full term pregnancy, HIV (+ or -), human immunodeficiency virus (positive or negative); HL-VMB, high lactobacillus vaginal microbiome; (HR) HPV, (high risk) human papillomavirus; HSIL, high-grade squamous intraepithelial lesion; IBD, inflammatory bowel disease; ICC, invasive cervical cancer; IUD, intrauterine device; IVF, in vitro fertilisation; LL-VMB, low lactobacillus vaginal microbiome; LSIL, low-grade squamous intraepithelial lesion; LCP: Lactobacillus crispatus predominant; LIP: Lactobacillus iners predominant; LP: lactobacillus predominant; NA, not available; NP, not pertinent (because the estimated is larger than the observed, and there is no evidence of excess statistical significance based on the assumption made for the plausible effect size); OC: oral contraception; SCC, squamous cell carcinoma; SIL, squamous intraepithelial lesion; SLE, systemic lupus erythematosus, VMB: vaginal microbiome

Key: [a] Number of studies, [b] Sum of all study cohorts in the meta-analysis, [c] Relative risk and 95% confidence interval of largest study (smallest standard error) in each meta-analysis, [d] Random effects refer to summary risk ratio (95% confidence interval) using the random-effects model, [e] P value of summary random effects estimate, [f] P value from the Egger's regression asymmetry test (p <0.10), [g] Expected number of statistically significant studies using the point estimate of the largest study (smallest standard error) as the plausible effect size, [h] Observed/Expected number of statistically significant studies, [i] P value of the excess statistical significance test (p <0.10)
All statistical tests were two-sided
