# Supplementary material for: Risk factors for human papillomavirus infection, cervical intraepithelial neoplasia and cervical cancer: an umbrella review and follow-up Mendelian randomisation studies
Source: BMC Med. 2023 Jul 27;21:274. doi: 10.1186/s12916-023-02965-w (PMC10375747; doi:10.1186/s12916-023-02965-w)
Supplement: Supplementary file 9 — Additional file 9: Supplementary Table 8. Excluded duplicate studies and studies selected in their place to be included in the analysis. [file 12916_2023_2965_MOESM9_ESM.pdf]

Table S8: Excluded duplicate studies and studies selected in their place to be included in the analysis.

Exposure	Exposure contrast	Author, year	Outcome	N[a]	N[b]	Summary RR & 95% CI[c]	Included	Evidence grade
BMI	Obese vs not	Poorolajal/Jenabi 2016	Cervical cancer incidence	7	0	1.03(0.81-1.25)	No	
BMI	Obese vs not	Poorolajal/Jenabi 2016	Cervical cancer incidence	2	2	1.1(1.03-1.17)	No	
BMI	Overweight vs not	Poorolajal/Jenabi 2016	Cervical cancer incidence	7	0	1.4(1.08-1.71)	No	
BMI	Overweight vs not	Poorolajal/Jenabi 2016	Cervical cancer incidence	2	2	1.06(0.6-1.52)	No	
BMI	Highest vs lowest	WCRF CUP 2018	Cervical cancer incidence	7	7	1.13(0.93-1.38)	Yes	NS
BMI	Highest vs lowest	WCRF CUP 2018	Cervical cancer incidence	4	4	1.82(1.07-3.10)	Yes	Weak
BMI	For 5kg/m2 increase	WCRF CUP 2018	Cervical cancer incidence	9	9	1.02(0.97-1.07)	Yes	NS
BMI	For 5kg/m2 increase	WCRF CUP 2018	Cervical cancer incidence	7	7	1.01(0.96-1.06)	Yes	NS
COCP	Current users, risk per year of use	Appleby 2007	Cervical cancer incidence	18	3	1.07(1.05-1.08)	Yes	NS
COCP	2-9 years since last use, risk per year of use	Appleby 2007	Cervical cancer incidence	18	3	1.03(1.01-1.04)	Yes	NS
COCP	10+ years since last use, risk per year of use	Appleby 2007	Cervical cancer incidence	18	3	0.98(0.97-1.00)	Yes	NS

Oral contraception	Ever vs never	Peng 2017	Cervical cancer incidence	16	0	1.12(0.9-1.38)	Yes	NS
Oral contraception	<5 years of use vs never	Peng 2017	Cervical cancer incidence	11	0	0.84(0.68-1.04)	No	
Oral contraception	5-10 years of use vs never	Peng 2017	Cervical cancer incidence	8	0	1.06(0.65-1.71)	No	
Oral contraception	>10 years of use vs never	Peng 2017	Cervical cancer incidence	9	0	1.25(0.76-2.06)	No	
Oral contraception	ever vs never	Rodriguez 1992	Cervical dysplasia incidence	20		1.31(1.24-1.38)	No	
Oral contraception	ever vs never	Rodriguez 1992	Carcinoma in situ incidence	28		1.29(1.18-1.41)	No	
Oral contraception	ever vs never	Rodriguez 1992	Invasive cervical cancer incidence	16		1.13(0.99-1.27)	No	
Oral contraception	ever vs never	Gierisch 2013	Cervical cancer incidence	11	4	1.21(0.91-1.61)	No	
Oral contraception	<5 ys user vs never	Smith 2003	Cervical cancer incidence	20	4	1.10(1.00-1.20)	Yes	Weak
Oral contraception	5-9 ys user vs never	Smith 2003	Cervical cancer incidence	21	4	1.60(1.40-1.70)	Yes	Weak
Oral contraception	>/=10 ys user vs never	Smith 2003	Cervical cancer incidence	13	3	2.20(1.90-2.40)	Yes	Weak
Oral contraception	<5 ys user vs never	Berrington 2004	Adenocarcinoma (invasive and in situ) incidence	6	0	1.33(1.09-1.62)	No	
Oral contraception	5-9 ys user vs never	Berrington 2004	Adenocarcinoma (invasive and in situ) incidence	6	0	1.60(1.19-2.15)	No	
Oral contraception	10+ ys user vs never	Berrington 2004	Adenocarcinoma (invasive and in situ) incidence	4	0	2.19(1.58-3.02)	No	
Oral contraception	<5 ys user vs never	Berrington 2004	SCC (invasive and in situ) incidence	6	0	1.11(1.01-1.22)	No	
Oral contraception	5-9 ys user vs never	Berrington 2004	SCC (invasive and in situ) incidence	6	0	1.51(1.29-1.77)	No	
Oral contraception	10+ ys user vs never	Berrington 2004	SCC (invasive and in situ) incidence	4	0	2.00(1.72-2.37)	No	
Oral contraception, <5 years of use	Current and recent users (<8 ys since last use) vs never	Smith 2003	Cervical cancer incidence	4	1	1.40(1.20-1.50)	Yes	NS

Oral contraception, <5 years of use	Past users (>8 ys since last use) vs never	Smith 2003	Cervical cancer incidence	4	0	1.10(1.00-1.20)	Yes	NS
Oral contraception, >5 years of use	Current and recent users (<8 ys since last use) vs never	Smith 2003	Cervical cancer incidence	4	0	2.10(1.80-2.40)	Yes	Weak
Oral contraception, >5 years of use	Past users (>8 ys since last use) vs never	Smith 2003	Cervical cancer incidence	4	0	1.40(1.10-1.90)	Yes	NS
Oral contraception and HPV+ve	<5 ys user vs never	Smith 2003	Cervical cancer incidence	5	2	0.90(0.70-1.20)	Yes	NS
Oral contraception and HPV+ve	5-9 ys user vs never	Smith 2003	Cervical cancer incidence	5	2	1.30(1.00-1.90)	Yes	NS
Oral contraception and HPV+ve	>/=10 ys user vs never	Smith 2003	Cervical cancer incidence	3	2	2.50(1.60-3.90)	Yes	NS
Injectable contraception	<5 ys user vs never	Smith 2003	Cervical cancer incidence	3	0	1.00(0.90-1.20)	Yes	Strong
Injectable contraception	>/=5 ys user vs never	Smith 2003	Cervical cancer incidence	2	0	1.20(1.00-1.60)	Yes	Weak
Parity	1-2 births vs P0	Berrington 2004	Adenocarcinoma (invasive and in situ) incidence	3	0	0.76-1.41	No	
Parity	3+ births vs P0	Berrington 2004	Adenocarcinoma (invasive and in situ) incidence	3	0	1.02-2.22	No	
Parity	1-2 births vs P0	Berrington 2004	SCC (invasive and in situ) incidence	3	0	1.10-1.40	No	
Parity	3+ births vs P0	Berrington 2004	SCC (invasive and in situ) incidence	3	0	2.08-3.53	No	
Parity	By increase of 1 FTP	Appleby 2006	Invasive cervical cancer incidence	17	1	1.08-1.12	Yes	Highly suggestive
Parity, age at first FTP	By 1-year decrease	Appleby 2006	Invasive cervical cancer incidence	17	1	1.06-1.09	Yes	Highly suggestive
Parity	By increase of 1 FTP	Appleby 2006	CIN3 incidence	15	5	1.03-1.09	Yes	NS
Parity, age at first	By 1-year decrease	Appleby 2006	CIN3 incidence	15	5	1.03-1.06	Yes	NS

FTP								
Parity	Pregnant vs non-pregnant	Liu 2014	HPV incidence	14	14	1.25-1.61	Yes	weak
Parity	By increase of 1 FTP	Appleby 2006	Invasive cervical cancer incidence	20	4	1.08-1.12	Yes	NS
Parity	By increase of 1 FTP	Appleby 2006	CIN3 incidence	17	7	1.03-1.09	Yes	NS
Parity, age at first FTP	1-year decrease in age at FTP	Appleby 2006	Invasive cervical cancer incidence	20	4	1.06-1.09	Yes	NS
Parity, age at first FTP	1-year decrease in age at FTP	Appleby 2006	CIN3 incidence	17	7	1.03-1.06	Yes	NS
Parity, age at first birth	20-24 vs 25+ ys	Berrington 2004	Adenocarcinoma (invasive and in situ) incidence	3	0	0.80-1.46	No	
Parity, age at first birth	20-24 vs 25+ ys	Berrington 2004	Adenocarcinoma (invasive and in situ) incidence	3	0	0.73-1.48	No	
Parity, age at first birth	</= 19 vs 25+ ys	Berrington 2004	SCC (invasive and in situ) incidence	3	0	0.83-1.25	No	
Parity, age at first birth	</= 19 vs 25+ ys	Berrington 2004	SCC (invasive and in situ) incidence	3	0	1.01-1.59	No	

Abbreviations: HPV: human papilloma virus; RR: relative risk, CI: confidence interval, COCP: combined oral contraceptive pill; ys: years; +ve: positive; SCC: squamous cell carcinoma; P0: parity 0; FTP: full term pregnancy, CIN: cervical intraepithelial neoplasia.

Key: [a] Number of studies, [b] Number of cohort studies, [c] Summary relative risk of random effects model including all study types
