# Supplementary material for: Risk factors for human papillomavirus infection, cervical intraepithelial neoplasia and cervical cancer: an umbrella review and follow-up Mendelian randomisation studies
Source: BMC Med. 2023 Jul 27;21:274. doi: 10.1186/s12916-023-02965-w (PMC10375747; doi:10.1186/s12916-023-02965-w)
Supplement: Supplementary file 10 — Additional file 10: Supplementary Table 9. Mendelian randomisation (MR) analysis; exposure-outcome pairs included in the main analysis, cohorts only. [file 12916_2023_2965_MOESM10_ESM.pdf]

Table S9: Mendelian randomisation (MR) analysis; exposure-outcome pairs included in the main analysis, cohorts only.

	Exposure	Example of Exposure contrast	Outcome	MR
1.	Age in pregnancy	Per one-year decrease in age at full term pregnancy	CIN3 incidence	OK
2.	Alcohol intake	Highest vs lowest	CC incidence	OK
3.	BMI	Highest vs lower	CC mortality	OK
4.	Candida Albicans	Yes vs no	CIN incidence	Weak instrument
5.	Chlamydia trachomatis	Yes vs no	CIN1+ incidence	No GWAS available
6.	Contraception, oral	Short duration <5 years user vs never	CIN3+ incidence	Weak instrument
7.	Environmental tobacco smoke exposure	Increased vs lower	CIN2+ incidence	OK
8.	Fruit intake	Highest vs lowest (no HPV adjustment)	Cervical dysplasia	Weak instrument
9.	GDM	Yes vs no	CC incidence	OK
10.	Height	Height per 5 cm increase	CC incidence	OK
11.	HIV	positive vs negative	HR-HPV incidence	No female specific GWAS
12.	HIV positive	On ART vs non-ART users	SIL-CIN regression	No female specific GWAS
13.	IBD on immunosuppression	IBD on immunosuppression vs health controls	High grade dysplasia/cancer incidence	OK
14.	IVF	Yes vs no	CC incidence	No GWAS available
15.	Parity	Per increase of 1 full term pregnancy	CIN3 incidence	OK
16.	Retinoid use	Yes vs no	complete regression of CIN2 at 9-27 months	No GWAS available
17.	Sexual partners	Multiple vs few partners	CIN incidence	OK
18.	Smoking	Yes vs no	HPV incidence	OK
19.	Pregnant	Yes vs no	HPV incidence	No GWAS available
20.	Rheumatoid arthritis	Yes vs no	CC incidence	OK
21.	Vaginosis, BV	Yes vs no	CIN prevalence	No GWAS available

22.	Vegetable intake	Highest vs lowest (no HPV adjustment)	Cervical Dysplasia	Weak instrument

Abbreviations: CIN: cervical intraepithelial neoplasia, CC: cervical cancer, BMI: body mass index, GWAS: genome-wide associations studies, HPV: human papillomavirus, GDM: gestational diabetes, HIV: human immunosufficiency virus, HR-HPV: high risk human papillomavirus, ART: antiretroviral treatment, SIL: squamous intraepithelial lesion, IBD: inflammatory bowel disease, IVF: in vitro fertilisation, BV: bacterial vaginosis
