# Supplementary material for: Risk factors for human papillomavirus infection, cervical intraepithelial neoplasia and cervical cancer: an umbrella review and follow-up Mendelian randomisation studies
Source: BMC Med. 2023 Jul 27;21:274. doi: 10.1186/s12916-023-02965-w (PMC10375747; doi:10.1186/s12916-023-02965-w)
Supplement: Supplementary file 11 — Additional file 11: Supplementary Table 10. Main GWAS study characteristics. [file 12916_2023_2965_MOESM11_ESM.pdf]

Table S10. Main GWAS study characteristics.

Exposure	Units	Total Sample Size	Exposure N (Ukbb)*	Outcome N (Ukbb)*	Sample overlap*	First author	Pubmed	Characteristics	Source of GWAS
Lifetime smoking index	Lifetime smoking score (per unit, approx.1 pack/day for 10 years)	462690	462690	273377	0.59	Wootton	31689377	All	Ukbb
Age at first pregnancy	Age (years)	189656	40082	273377	0.15	Barban	27798627	Women only	MA of 62 studies
Number of sexual partners	Lifetime number of sexual partners	370711	370711	273377	0.74	Karlsson -Linner	30643258	All	MA of 7 studies
Systemic lupus erythematosis	Case/control	7,219 cases and 15,991 controls	0	273377	0.00	Bentham	26502338	All	HRS cohort
Rheumatoid arthritis	Case/control	29,880 cases and 73,758 controls	0	273377	0.00	Okada	24390342	All	MA of 22/18 studies
Inflammatory bowel disease	Case/control	25,042 cases and 34,915 controls	0	273377	0.00	DeLange	28067908	All	GWAS + IIBDGC MA
Alcohol intake	Alcohol intake (units per week)	59088	59088	273377	0.22	Clarke	28937693	Women only	Ukbb
Body Mass Index (BMI)	BMI (per SD, approx. 5.2 kg/m2)	434794	262817	273377	0.96	Pulit	30239722	Women only	Ukbb + GIANT MA
Gestational Diabetes Mellitus (GDM)	Case/control	1180 cases and 4939 controls	6119	273377	0.02	Neale's lab	-	Women only	Ukbb (neale's lab)
Height	Standing height (rank-normalized)	193785	193785	273377	0.71	Neale's lab	-	Women only	Ukbb (neale's lab)
Parity	Number of live births (rank-normalized)	193953	193953	273377	0.71	Neale's lab	-	Women only	Ukbb (neale's lab)

**Approximate sample overlap between the exposure and the outcome GWAS, based on the inclusion of UK biobank participants.*
