# Supplementary material for: Risk factors for human papillomavirus infection, cervical intraepithelial neoplasia and cervical cancer: an umbrella review and follow-up Mendelian randomisation studies
Source: BMC Med. 2023 Jul 27;21:274. doi: 10.1186/s12916-023-02965-w (PMC10375747; doi:10.1186/s12916-023-02965-w)
Supplement: Supplementary file 12 — Additional file 12: Supplementary Table 11. Two-sample inverse variance weighted mendelian randomization full results of the analyses of risk factors on cervical cancer. [file 12916_2023_2965_MOESM12_ESM.pdf]

Table S11. Two-sample inverse variance weighted mendelian randomization full results of the analyses of risk factors on cervical cancer.

Exposure	OR	SE	Lower 95%CI	Upper 95%CI	P	FDR
Lifetime smoking index	2.456	0.509	1.636	3.687	1.48E-05	0.00008
Age at first pregnancy	0.800	0.068	0.676	0.945	8.79E-03	0.02417
Number of sexual partners	1.948	0.300	1.440	2.634	1.49E-05	0.00008
Systemic lupus erythematosis	0.987	0.015	0.958	1.016	3.73E-01	0.62523
Rheumatoid arthritis	1.098	0.027	1.046	1.153	1.75E-04	0.00064
Inflammatory bowel disease	0.985	0.023	0.940	1.032	5.12E-01	0.62523
Alcohol consumption	0.947	0.473	0.375	2.395	9.09E-01	0.90899
Body mass index	1.051	0.063	0.929	1.190	4.28E-01	0.62523
Gestational diabetes mellitus	0.912	0.458	0.372	2.237	8.41E-01	0.90899
Parity	0.384	0.696	0.098	1.505	1.70E-01	0.37370
Height	0.977	0.035	0.913	1.046	5.08E-01	0.62523

Abbreviations: FDR: false discovery rate; OR: odds ratio; P: P-value; SE: standard error; 95%CI: 95% confidence interval.
