# Supplementary material for: Risk factors for human papillomavirus infection, cervical intraepithelial neoplasia and cervical cancer: an umbrella review and follow-up Mendelian randomisation studies
Source: BMC Med. 2023 Jul 27;21:274. doi: 10.1186/s12916-023-02965-w (PMC10375747; doi:10.1186/s12916-023-02965-w)
Supplement: Supplementary file 13 — Additional file 13: Supplementary Table 12. Results from Mendelian randomisation sensitivity analyses of risk factors on cervical cancer. Supplementary Table 13. Results from multivariable (MV) Mendelian randomisation (MR) sensitivity analyses of risk factors on cervical cancer. [file 12916_2023_2965_MOESM13_ESM.pdf]

Table S12. Results from Mendelian randomisation sensitivity analyses of risk factors on cervical cancer.

	Weighted Median				MR-Egger					MR-PRESSO			Power achieved**		
	OR	CI-lower	CI-upper	P-value	P-value Intercept	OR	CI-lower	CI-upper	P-value	OR (Corrected)	SE (Corrected)	P-value (Corrected)	N_instr	R2***	power
**Lifetime smoking index**	1.992	1.152	3.445	0.014	0.033	0.457	0.092	2.263	0.337	2.314	0.460	0.000	125	0.005	1.000
**Number of sexual partners**	1.945	1.301	2.906	0.001	0.229	5.010	1.043	24.066	0.044	N.O.*	N.O.	N.O.	86	0.010	1.000
**Rheumatoid arthritis**	1.169	1.099	1.244	0.000	0.008	1.201	1.108	1.301	0.000	1.078	0.025	0.002	56	0.062	0.380
**Age at first pregnancy**	0.813	0.659	1.004	0.054	0.707	0.411	0.013	13.219	0.615	N.O.	N.O.	N.O.	6	0.025	0.585
**Systemic lupus erythematosis**	0.986	0.954	1.019	0.394	0.020	0.927	0.873	0.984	0.012	1.001	0.011	0.930	45	0.186	0.068
**Inflammatory bowel disease**	1.023	0.962	1.088	0.467	0.387	1.040	0.910	1.189	0.561	N.O.	N.O.	N.O.	131	0.090	0.062
**Alcohol consumption**	0.867	0.333	2.257	0.769	0.363	13.5	0.041	4479	0.380	N.O.	N.O.	N.O.	6	0.002	0.052
**Body mass index**	1.070	0.876	1.308	0.507	0.482	1.176	0.840	1.645	0.345	N.O.	N.O.	N.O.	311	0.049	0.120
**Gestational diabetes mellitus**	-	-	-	-	-	-	-	-	-	N.O.	N.O.	N.O.	2	0.010	0.093
**Parity**	0.668	0.167	2.669	0.569	0.001	0.000	0.000	0.000	0.000	N.O.	N.O.	N.O.	9	0.001	0.244
**Height**	0.914	0.828	1.009	0.074	0.276	0.901	0.767	1.059	0.205	N.O.	N.O.	N.O.	431	0.188	0.103

*N.O.: MR-PRESSO outlier test non significant.

**Using odds ratios from the main MR-IVW analysis, alpha = 0.05, and outcome sample size = 150 314 (4 769 cases)

***Approximate proportion of phenotypic variance explained by the included SNPs

Table S13. Results from multivariable (MV) Mendelian randomisation (MR) sensitivity analyses of risk factors on cervical cancer.

	OR	SE	CI-lower	CI-upper	P-value
Age at first pregnancy (univariable MR)	0.800	0.068	0.676	0.945	0.009
Age at first pregnancy (MV MR adj. for Number of sexual partners)*	0.720	0.065	0.634	0.818	0.007
Age at first pregnancy (MV MR adj. for Lifetime Smoking Index)*	0.709	0.217	0.463	1.086	0.189
Number of sexual partners (univariable MR)	1.948	0.300	1.440	2.634	1.49E-05
Number of sexual partners (MV MR adj. for Lifetime Smoking Index)*	1.879	0.333	1.328	2.661	3.74E-04
Lifetime smoking index (MV MR adj. for Number of sexual partners)*	1.737	0.425	1.075	2.806	2.42E-02

*Instruments selected based on exposure.
